# Ciguatoxicity of *Gambierdiscus* and *Fukuyoa* species from the Caribbean and Gulf of Mexico

**DOI:** 10.1371/journal.pone.0185776

**Published:** 2017-10-18

**Authors:** R. Wayne Litaker, William C. Holland, D. Ransom Hardison, Francesco Pisapia, Philipp Hess, Steven R. Kibler, Patricia A. Tester

**Affiliations:** 1 National Ocean Service, National Centers for Coastal Ocean Science, Center for Coastal Fisheries and Habitat Research, Beaufort, North Carolina, United States of America; 2 L'Institut Français de Recherche pour l'Exploitation de la Mer, Laboratoire Phycotoxines, Nantes, France; 3 Ocean Tester, LLC, Beaufort, North Carolina, United States of America; University of Connecticut, UNITED STATES

## Abstract

Dinoflagellate species belonging to the genera *Gambierdiscus* and *Fukuyoa* produce ciguatoxins (CTXs), potent neurotoxins that concentrate in fish causing ciguatera fish poisoning (CFP) in humans. While the structures and toxicities of ciguatoxins isolated from fish in the Pacific and Caribbean are known, there are few data on the variation in toxicity between and among species of *Gambierdiscus* and *Fukuyoa*. Quantifying the differences in species-specific toxicity is especially important to developing an effective cell-based risk assessment strategy for CFP. This study analyzed the ciguatoxicity of 33 strains representing seven *Gambierdiscus* and one *Fukuyoa* species using a cell based Neuro-2a cytotoxicity assay. All strains were isolated from either the Caribbean or Gulf of Mexico. The average toxicity of each species was inversely proportional to growth rate, suggesting an evolutionary trade-off between an investment in growth versus the production of defensive compounds. While there is 2- to 27-fold variation in toxicity within species, there was a 1740-fold difference between the least and most toxic species. Consequently, production of CTX or CTX-like compounds is more dependent on the species present than on the random occurrence of high or low toxicity strains. Seven of the eight species tested (*G*. *belizeanus*, *G*. *caribaeus*, *G*. *carolinianus*, *G*. *carpenteri*, *Gambierdiscus* ribotype 2, *G*. *silvae* and *F*. *ruetzleri*) exhibited low toxicities, ranging from 0 to 24.5 fg CTX3C equivalents cell^-1^, relative to *G*. *excentricus*, which had a toxicity of 469 fg CTX3C eq. cell^-1^. Isolates of *G*. *excentricus* from other regions have shown similarly high toxicities. If the hypothesis that *G*. *excentricus* is the primary source of ciguatoxins in the Atlantic is confirmed, it should be possible to identify areas where CFP risk is greatest by monitoring only *G*. *excentricus* abundance using species-specific molecular assays.

## Introduction

Species in the dinoflagellate genera *Gambierdiscus* and *Fukuyoa* produce cyclic polyether toxins known as ciguatoxins (CTXs) and maitotoxins (MTXs). These compounds are among the most potent naturally occurring toxins known [1]. CTXs activate voltage-gated sodium channels and disrupt normal cellular function, with nerve cells being particularly susceptible [2–6]. These toxins are lipophylic and accumulate in the food webs of many tropical, shallow water marine ecosystems reaching their highest concentrations in fish [7–9]. The consumption of fish containing sufficient CTX results in an illness known as ciguatera fish poisoning (CFP) in humans. It is the most common non-bacterial seafood-related illness and characterized by a variety of gastrointestinal and neurological symptoms, and on rare occasions, death [10, 11]. This illness is not only a concern for local populations in the tropics dependent on fish as a protein source, but also for consumers of reef fish worldwide [12, 13]. There is concern that increasing ocean temperatures in coming decades may promote range extensions of CTX-producing dinoflagellates into higher latitudes not currently impacted by CFP [14, 15]. This range expansion is supported by recent studies documenting the occurrence of *Gambierdiscus* species in more temperate waters surrounding the main islands of Japan, the Mediterranean Sea, the Canary Islands and along the eastern coasts of North and South America [14–23].

While only some *Gambierdiscus* and *Fukuyoa* isolates produce CTX or CTX-like compounds as measured by mouse, cytotoxicity or LC-MS assays, most produce varying amounts of water soluble MTXs (S1 Table). Though MTXs are slightly more toxic than CTXs when measured by mouse bioassay using intra-peritoneal injections, they are only found in the digestive tract and liver of fish, and are unlikely to contribute to CFP unless these tisses are consumed [24–26]. Consequently, this study focused on characterizing CTX toxicity among *Gambierdiscus* and *Fukuyoa* species as these toxins pose the predominant threat to human health.

Currently there is no systematic screening protocol for testing fish for ciguatoxins. This is due largely to the expense of running the analytical assays and the limited availability of certified standards [27]. Given this situation, estimating CFP risk is problematic. CFP frequently occurs in tropical archipelagos well away from metropolitan centers, so the ability to test for the toxins is limited. One approach for estimating CFP risk is to develop a cell abundance-based monitoring effort to guide the need for toxin measurements. For this approach to be effective, it is necessary that fluxes of toxins into the food web be proportional to the abundances of *Gambierdiscus* and *Fukuyoa* species [8]. The data from a five-year survey in the Pacific by Chinain et al. [28] indicate this is not necessarily the case there. While the two years with the highest *Gambierdiscus* abundances exhibited higher than normal toxicity, across all years the relationship between *Gambierdiscus* abundance and measured toxicity was poor. Chinain et al. [28] hypothesized the variation was due to the presence of more toxic isolates or species whose relative abundances varied from year to year. Subsequent studies in the Pacific demonstrated that *G*. *polynesiensis* was considerably more toxic than the other species tested; suggesting changes in the relative abundance of just one species may significantly increase the CFP risk [29, 30]. The extent to which similarly toxic species or strains occur in the Caribbean and Gulf of Mexico (GOM) is the topic of this study. Thirty-three strains representing eight species of *Gambierdiscus* or *Fukuyoa* from the Caribbean were assessed using the cell based neuro-2a assay (CBA-N2a). The results showed *G*. *excentricus* had much higher toxicity than other co-occurring *Gambierdiscus* or *Fukuyoa* species, indicating it may be the dominant producer of CTX or CTX-like compounds in the Caribbean and GOM.

## Materials and methods

### Ethics statement

The material in this manuscript has not been published in whole or in part elsewhere nor is currently being considered for publication in another journal. All the authors have been personally and actively involved in substantive work leading to the manuscript, and will hold themselves jointly and individually responsible for its content. This research used only isolates of microalgal species belonging to the genera *Fukuyoa* and *Gambierdiscus*. No human or animal subjects were involved and no collection permits were required.

### Strain and culture conditions

Strains of seven *Gambierdiscus* (*G*. *belizeanu*s n = 6, *G*. *caribaeus* n = 7, *G*. *carolinianus* n = 5, *G*. *carpenteri* n = 5, *G*. *excentricus* n = 1, *Gambierdiscus* ribotype 2 n = 5, *G*. *silvae* n = 1) and one *Fukuyoa* (*F*. *ruetzleri* n = 3) species obtained from the Caribbean and GOM were used to determine specific growth rates and toxicity. Four of the strains (CCMP 1655, CCMP 399, CCMP 1733, and CCMP 1651) were obtained from the National Centre for Marine Algae and Microbiota (East Boothbay, Maine, USA). All other strains were established as single cell isolates from field material as described previously [31] (Table 1). Where possible, isolates of the species tested were selected from geographically disparate locations.

**Table 1:** The species, strain designations, isolate locations, replicate growth rates and toxicities of the *Gambierdiscus* and *Fukuyoa* strains examined in this study. The citations in the species column indicate where the species was described. The reference(s) under the strain designation indicate other publications where the strain has been studied. Many of the strains analyzed for CTX-like activity in this study were also assayed for maitotoxicity in separate investigations [32, 33]. The strain growth rates (± standard deviation) were determined from triplicate, independent cultures started for each isolate. Mean species growth rates and average toxicities were determined by averaging all replicate culture data for a given species. Toxicity was normalized both as femtograms (fg) CTX3C equivalents [eq.] cell^-1^ and per biovolume attograms (ag) CTX3C eq. μm^-3^. Numbers in parentheses in the data cells of the last three columns = coefficient of variation. Correlation coefficients (R^2^) for the time versus cell number relationships used to the calculate growth rates for each of the cultures exceeded 0.98.

Species	Strain	Origin	Growth rate (d^-1^)	Toxicity (fg CTX3C eq. cell^-1^)	Biovolume (μm^-3^)	Toxicity (ag CTX3C eq. μm^-3^)	Average Within Species Growth rate (d^-1^)	Average Within Species Toxicity (fg CTX3C eq. cell^-1^)	Average Within Species Toxicity (ag CTX3C eq. μm^-3^)
*F*. *ruetzleri* [31,34]	Gam 1 [32, 35]	Carrie Bow Cay, Belize	0.17±0.003	24.50±0.12	66,635	0.37±0.002	0.18±0.003 (2%)	10.6±12.4 (116%)	0.16±0.019 (119%)
	NC YASU 7–21 [this study]	Continental shelf off North Carolina, USA	0.18±0.001	0.88±0.40	71,877	0.012±0.006			
	WH55-Gam 4 [33]	Flower Garden Banks National Marine Sanctuary (West Bank), Gulf of Mexico, USA	0.17±0.001	6.50±1.47	75,757	0.086±0.020			
*G*. *belizeanus* [7]	CCMP399 [15, 31, 33, 35]	St. Barthelemy, Collectivity of France, Caribbean	0.20±0.001	0.65±0.10	100,663	0.0065±0.001	0.18±0.026 (14%)	0.85±0.81 (105%)	0.0072±0.006 (83%)
	Keys Gam 1 [32, 33]	Florida Keys, USA	0.18±0.001	0.54±0.03	107,489	0.0050±0.00028			
	St. Maarten Gam 12 [this study]	St. Maarten, Collectivity of France, Caribbean	0.17±0.001	0.49±0.26	109,449	0.0045±0.0024			
	ST1 Gam F4 [32]	St. Thomas, US Virgin Islands	0.13±0.001	0.43±0.38	135,352	0.0032±0.0028			
	SW Gam 2 [32]	Southwater Cay, Belize	0.15±0.001	2.49±1.28	128,576	0.019±0.01			
	Turks Gam 2 [this study]	The Turks and Caicos, Atlantic	0.18±0.001	0.51±0.16	107,206	0.0045±0.0015			
*G*. *caribaeus* [31]	CBC Gam 1 [32]	Carrie Bow Cay, Belize	0.16±0.004	0.62±0.12	243,981	0.0025±0.00049	0.17±0.024 (14%)	0.66±0.34 (52%)	0.003±0.0016 (53%)
	CCMP1651 [15, 31, 32, 36]	Grand Cayman Island, Caribbean	0.17±0.003	0.48±0.04	188,470	0.0026±0.0002			
	CCMP1733 [31–33, 37]	Carrie Bow Cay, Belize	0.16±0.002	0.80±0.43	214,306	0.0037±0.0020			
	Dive 1 FA [32, 33]	Carrie Bow Cay, Belize	0.17±0.001	0.69±0.19	237,758	0.0029±0.0008			
	Keys Jar 7 [32]	Florida Keys, USA	0.22±0.002	0.19±0.03	248,385	0.00077±0.000121			
	Mexico Algae 1 [33]	Cancun, Mexico	0.17±0.001	1.29±0.40	212,313	0.0061±0.0019			
	SW Gam5 [32]	Southwater Cay, Belize	0.15±0.003	0.52±0.26	240,568	0.0022±0.0011			
*G*. *carolinianus* [31]	Bill Aruba Gam 5 [this study]	Aruba, Caribbean	0.15±0.003	1.03±0.94	155,657	0.0066±0.0060	0.17±0.017 (10%)	0.27±0.43 (162%)	0.0018±0.0028 (156%)
	Jamaica algae 2 Gam 22 [this study]	Ocho Rios, Jamaica, Caribbean	0.18±0.005	0.10±0.03	147,450	0.00068±0.00020			
	Lobster Rock N7 [32]	Continental shelf off North Carolina, USA	0.19±0.003	ND	179,310	0			
	RROV5 [32, 33]	Puerto Rico, USA	0.19±0.004	0.02±0.01	160,763	0.00012±.000062			
	St. Maarten Gam 5 [this study]	St. Maarten, Caribbean	0.16±0.004	0.18±0.06	108,013	0.0017±0.00056			
*G*. *carpenteri* [31]	Bill Aruba Gam 15 [this study]	Aruba, Caribbean	0.13±0.002	0.71±0.21	130,630	0.0054±0.0016	0.163±0.026 (16%)	0.89±0.41 (47%)	0.0045±0.0018 (45%)
	GT4 [32, 33, 35]	Carrie Bow Cay, Belize	0.20±0.003	0.29±0.16	179,522	0.0016±0.00089			
	Jamaica Algae 2 Gam 1 [32, 33]	Ocho Rios, Jamaica, Caribbean	0.17±0.002	0.93±0.17	204,140	0.0046±0.00083			
	Mexico Algae 2 Gam 1 [this study]	Cancun, Mexico	0.15±0.003	1.14±0.18	206,306	0.0055±0.00087			
	WBHR21 [32, 33]	Flower Garden Banks National Marine Sanctuary (West Bank), Gulf of Mexico, USA	0.18±0.004	1.37±0.30	254,476	0.0054±0.0012			
*G*. *excentricus* [38]	Pulley Ridge Gam 2 [33]	Pulley Ridge, Florida, USA	0.057±0.002	469±10	179,522	2.61±0.00557	0.057	469	2.61
*Gambierdiscus* ribotype 2 [31]	CCMP1655 [15, 32, 33, 36]	Martinique, Caribbean	0.15±0.001	10.9±0.36	147,700	0.074±.0024	0.128±0.010 (8%)	6.62±2.52 (38%)	0.046±0.016 (35%)
	Mixed PR Gam 4 [32, 33]	Puerto Rico, USA	0.12±0.001	6.63±0.54	139,322	0.048±0.0039			
	SJ3 Gam F2 [32]	St. John, US Virgin Islands	0.12±0.001	4.99±0.38	143,511	0.035±0.0027			
	St. Maarten Gam 10 [33]	St. Maarten, Collectivity of France, Caribbean	0.141±0.002	4.66±1.01	141,570	0.033±0.0071			
	SW Algae Gam 1 [33]	Southwater Cay, Belize	0.13±0.001	5.90±0.80	143,209	0.041±0.0056			
*G*. *silvae* [20]	Curacao Gam 11 [this study]	Curacao, Caribbean	0.098±0.024	19.6±4.21	70,028	0.28±0.060	0.98	19.6	0.28

Cells were cultured in a Percival Scientific incubator (Perry, IA, USA) maintained at 27°C with a 12:12 h light:dark cycle. Photosynthetically active radiation (PAR) was maintained at 90–100 μmol photons m^-2^ s^-1^ by horizontally mounted fluorescent lamps (Full Spectrum Solutions, Jackson, MI, USA). Light intensity was measured using a model QSL-100 4π wand meter (Biospherical Instruments Inc., San Diego, CA, USA).

Growth medium consisted of 0.2 μm filtered Gulf Stream seawater (salinity 33) in 250 mL tissue culture flasks with vented caps (BD Biosciences, Bedford, MA, USA). Vitamins and nutrients were added according to a modified K-medium protocol [39]. Phosphate was added in the form of Na_2_ *β*-glycerophosphoric acid, 5-hydrate at twice the concentration called for by K-medium protocol. An EDTA-trace metal buffer system was used with the omission of copper [40, 41]. Microwave treatment was used to sterilize the medium [42]. Culture pH was monitored using a Thermo Orion 3-Star pH meter with a Ross ultra-combination pH electrode (Thermo Fisher Scientific, Waltham, MA, USA) to ensure pH throughout experiments remained between 8.1 and 8.4. Cell densities were maintained at relatively low levels (100 to 1000 cells ml^-1^) to avoid nutrient or CO_2_ limitation.

### Growth rate analysis

**Fig 1:**
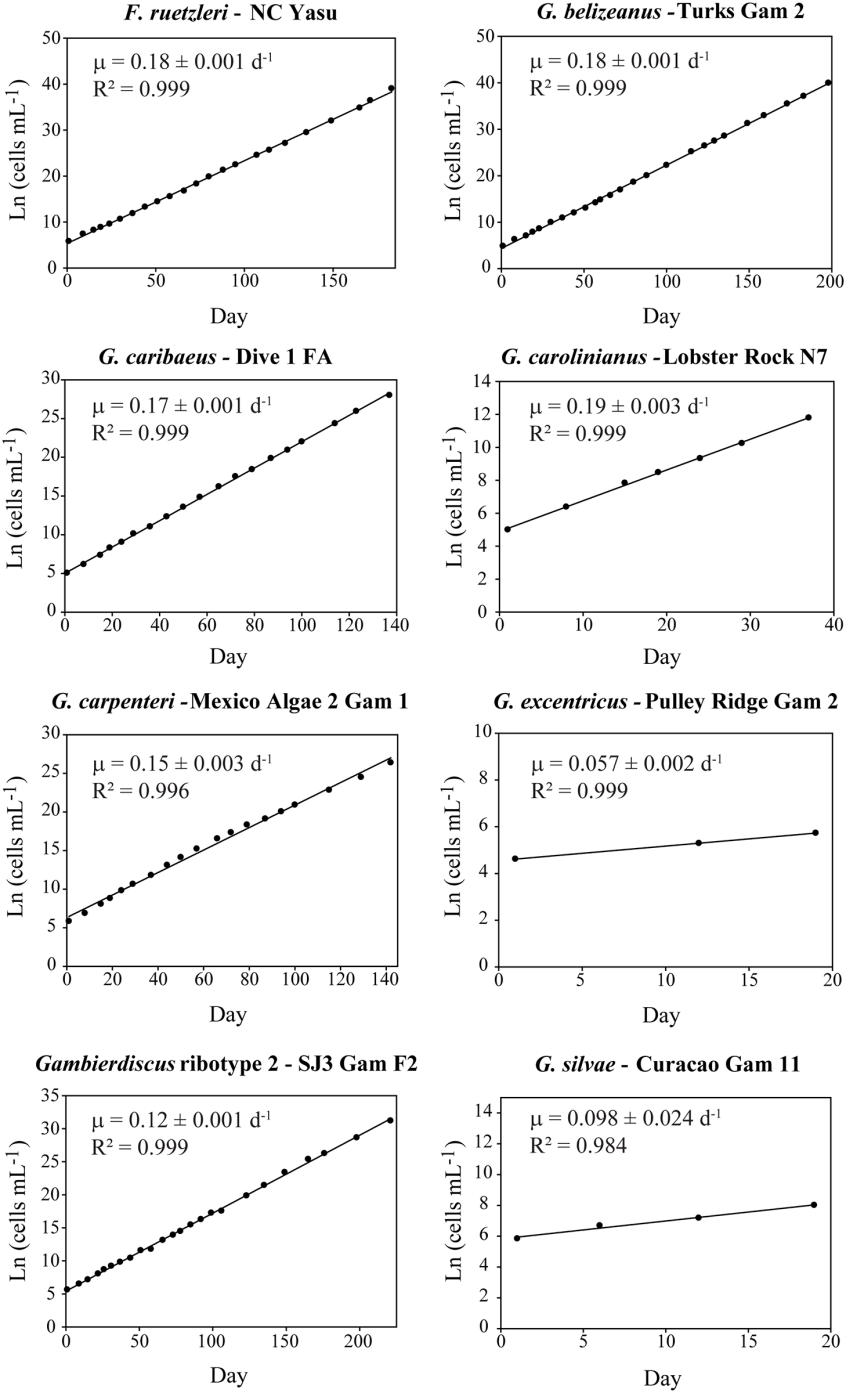
Representative plots showing the long-term steady state growth of the *Gambierdiscus* and *Fukuyoa* isolates achieved in this study. Exponential growth was achieved by acclimating cells to optimal temperature, light and nutrient conditions and maintained in exponential growth phase by periodic dilution with nutrient rich media.

For each isolate examined, three independent subcultures were established and the growth rate was determined for each. These batch subcultures were grown semi-continuously by removing calculated volumes based on cell density and adding fresh media to prevent cells from entering late log phase growth. Maximal steady state growth rates were maintained for the duration of each experiment, which ranged from a minimum of 18 days to a maximum of 200 days following a period of a month or more where cells were acclimated to exponential growth conditions. Cells were counted and their biovolume was measured every three to four days using a Beckman Coulter Multisizer^™^ 3 particle counter (Beckman Coulter Inc., Brea, CA) equipped with a 280 μm aperture and using 1.0 mL sample volumes. Samples were mixed thoroughly to ensure the cells were evenly distributed prior to counting. Specific growth rates (d^-1^) were calculated after accounting for dilutions using a linear regression of the ln cells mL^-1^ vs. time curve [41] (Fig 1). This specific growth rate method provides a better estimate of average growth rate than the common practice of choosing the three steepest growth points for a growth rate determination.

When cell densities were high enough, cells were harvested for toxicity by collecting a known number of cells on a 20 μm sieve and washing them with filtered seawater (Salinity = 33) into a 50 mL centrifuge tube. The cells were pelleted using centrifugation at 3200*g* for 10 min, the supernatant carefully decanted, and the pellet was processed immediately or stored at –20°C prior to extraction. Because ciguatoxicity varies with growth phase, the decision was made to ensure all the cultures were maintained in steady log phase growth prior to collection of cells for toxin analysis [29, 36, 43]. This assured that the intra-strain and inter-specific toxicity measurements were not biased due to harvesting cells in different growth phases.

### Reagents

All reagents used in this study were ACS grade or higher. Solvents were HPLC grade or higher purity. Pacific ciguatoxin-3C (CTX3C) was purchased from Wako Chemicals, USA, Inc. (Richmond, Virginia, USA) and provided by Institut Louis Malardé, Tahiti, French Polynesia (ILM). In this manuscript, we use the CTX nomenclature used by Yogi et al. [44] for the Pacific ciguatoxins (e.g. CTX3C rather than P-CTX-3C). References to Caribbean ciguatoxins are preceded by a C (e.g. C-CTX1). CTX3C standards were stored at –20°C and dissolved in 100% methanol prior to utilization in the CBA-N2a. All water used was Milli-Q Ultra-pure grade with 18.2 MΩ resistivity.

### Toxin extraction

Cell pellets were sonicated for 1 min in 100% methanol at 3 mL per 100,000 cells using a Qsonica, Q700 unit (Thermo Fisher Scientific Inc., Waltham, Massachusetts) with the tip amplitude setting at 50. Once cells were disrupted, the sample was centrifuged at 3,200g for 10 min and the supernatant was transferred to a 20 mL glass scintillation vial. This was repeated two more times and the methanol was collected and dried under N_2_ gas at 40°C. The dried extract was resuspended in dichloromethane (DCM) (5mL per 100,000 cells) and washed twice in a separatory funnel with 60:40 methanol:water (2.5 mL per 100,000 cells). The dichloromethane phases (bottom layer) were then collected and dried under N_2_ gas at 20°C. The dried extract was stored at –20°C. When ready to process, the DCM extract was resuspended in a volume of methanol that yielded a final concentration of 250–500 cells μL^-1^ [45].

### Neuro-2a cell based assay (CBA-N2a)

The CBA-N2a assay allows estimation of the concentration of CTXs or CTX-like compounds in extracts from fish or phytoplankton [38, 46–49]. The CBA-N2a assay measures bioactive compounds that bind voltage gated-sodium channels, not all of which are ciguatoxins [45]. Previous studies of *Gambierdiscus* and *Fukuyoa* species using LC-MS, and the same dichloromethane extraction protocol as this study, however, have shown CTX or "CTX-like" compound account for a majority of total cellular toxicity [29, 35, 43]. The consistency of these data support CTX or CTX-like compounds as the primary toxins measured in the isolates from this study.

The neuro-2a *Mus musculus* neuroblastoma cell line (N2a) used for the assay was obtained from the American Type Culture Collection (ATCC^®^ CCL-131^™^). Cells were grown and maintained in Eagle’s Minimum Essential Medium (EMEM; ATCC^®^ 30–2003) containing 2 mM L-glutamine, 1 mM sodium pyruvate, 100 μg mL^-1^ streptomycin, 100 units mL^-1^ penicillin, and 10% fetal bovine serum. Growth conditions were kept at 37°C using a humidified 5% CO_2_-enriched atmosphere. To prepare for toxicity analysis, the N2a cells were harvested with a trypsin-ethylenediaminetetraacetic acid (trypsin-EDTA) solution and seeded into each well of a 96-well microtiter plate at 30,000 cells per 100 μL of growth medium. The cells were subsequently incubated under the same growth conditions as above [36]. The plated N2a cells were allowed to settle and grow 20–24 h until they were >90% confluent at the bottom of each well. The standards, controls and samples were then added and the plates were incubated for 24 h. Each plate included control wells containing buffer only or buffer plus 5% methanol, the equivalent of the final methanol concentration when extracts were added. If the assay is working properly, both the buffer only and 5% methanol controls should contain a comparable number of live cells after the 24-hour incubation period. The CTX3C standard curves used in this assay ranged from 0.001–2,000 pg mL^-1^ and were suspended in the same 5% methanol buffer solution as the samples. Aliquots of each sample were added to six wells. Three of these wells contained 100 μM ouabain (O) and 10 μM veratridine (V) (O^+^/V^+^) to sensitize the CBA-N2a cells to CTX, and the other three contained no O/V (O^-^/V^-^). The O^-^/V^-^ wells served to identify other non-specific toxins present in the samples. Cell viability in the control wells, standard curve, sample O^-^/V^-^ and the O^+^/V^+^ wells were assessed after 20–24 hours of toxin exposure at 37°C using the colorimetric 3-(4,5-dimethylthiazol-2-yl)-2,5-diphenyl tetrazolium bromide (MTT) assay [49]. Cell mortality in the O^+^/V^+^ wells was converted to CTX estimates based on the CTX3C standard curve. The limit of detecton was 0.2 pg CTX3C eq. mL^-1^.

The resulting toxicity measurements were expressed as both femtogram CTX3C eq. per cell (fg cell^-1^) and attogram per μm^3^ cell volume (ag μm^-3^). The latter normalization employed the average cell volumes determined using the Multisizer when the cells were harvested. This approach determined if the variations in toxicity among isolates and among species were attributable to differences in cell size or toxicity per unit biomass.

For six of the eight species, multiple isolates were examined making it possible to estimate mean, standard deviaiton and coefficiants of variation in toxicity. To determine if the among species toxicity differences were statistically significant, a Kruskal-Wallis nonparametric one factor ANOVA was performed due to unequal variances. *Gambierdiscus excentricus* and *G*. *silvae* were excluded from the analysis because only a single clone was examined [50]. A Dunn’s test, which estimates median toxicities, was used to determine if species toxicities fell into distinct groups.

The extent of interspecific variation was also estimated by calculating the ratio between the average toxicitites for each species. In the case of *G*. *excentricus* and *G*. *silvae* the single toxicty estimate for each isolate was used to represent the mean value. Still another way to assess variations in toxicity used the mean growth rates and approximate toxicity per cell to estimate toxin production rates as fg CTX3C eq. cell^-1^ d^-1^. The results were plotted as species versus toxin production rates and the ratio of the least to the most prolific toxin producing species was calculated.

## Results

Five of the eight *Gambierdiscus* and *Fukuyoa* species studied had similar average growth rates ranging from 0.16 to 0.17 d^-1^. *Gambierdiscus* ribotype 2 (0.13 ± 0.01 d^-1^), *G*. *silvae* (0.098 d^-1^) and *G*. *excentricus* (0.057 d^-1^) grew more slowly (Fig 1) (Table 1). The observed growth rates were compared to those reported in other studies for the same species (S2 Table).

*Gambierdiscus excentricus* was the most toxic (469 fg CTX3C eq. cell^-1^) of the species examined (Table 1). The next most toxic species were *G*. *silvae* (19.6 fg CTX3C eq. cell^-1^) and *Gambierdiscus* ribotype 2 (4.7 to 10.9 fg CTX3C eq. cell^-1^). The remaining five species exhibited the following range of toxicities: *F*. *ruetzleri* (0.9 to 24.5 fg CTX3C eq. cell^-1^), *G*. *belizeanus* (0.4 to 2.5 fg CTX3C eq. cell^-1^); *G*. *caribaeus* (0.2 to 1.3 fg CTX3C eq. cell^-1^); *G*. *carolinianus* (non-detectable to 1.0 fg CTX3C eq. cell^-1^); and *G*. *carpenteri* (0.3 to 1.4 fg CTX3C eq. cell^-1^). The within species coefficient of variation in toxicity for the species where multiple isolates were tested ranged from 33% (*Gambierdiscus* ribotype 2) to 162% (*G*. *carolinianus*) (Table 1). Within a species, the highest toxicity isolate was ~2- to 27-fold more toxic than the least toxic isolate (S3 Table).

**Fig 2:**
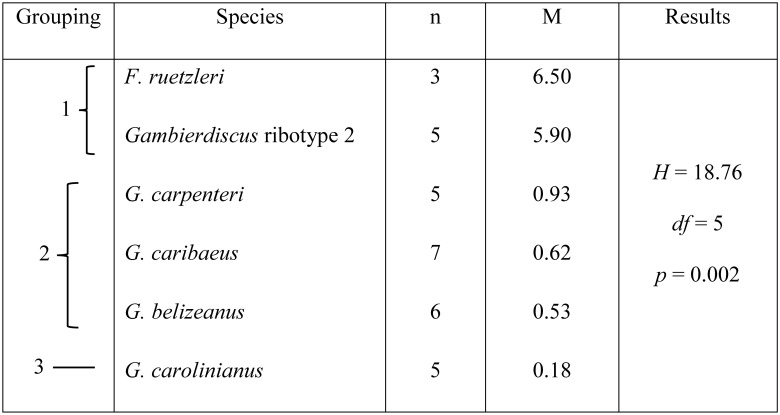
Results of a Kruskal-Wallis nonparametric one factor ANOVA for differences in CTX toxicity among *Gambierdiscus* and *Fukuyoa* species. *Gambierdiscus excentricus* and *G*. *silvae* were excluded from the analysis because only a single clone was examined. Abbreviations: n = sample size, M = median toxicity (fg CTX3C eq. cell^-1^), *H* = Kruskal-Wallis test statistic, *df* = degrees of freedom. Brackets denote result of the Dunn’s follow up test. The statistic is designed to estimate median toxicities to determine if the species partitioned into distinct groups.

The results of a one factor ANOVA (non-parametric Kruskal-Wallis test) using the species for which multiple isolates were available revealed toxicities among *F*. *ruetzleri*, *G*. *belizeanus*, *G*. *caribaeus*, *G*. *carolinianus*, *G*. *carpenteri* and *Gambierdiscus* ribotype 2 were significantly different (*H* = 18.76, *p* = 0.002) (Fig 2). A Dunn’s test indicated the six species were divided into three groups according to their median toxicities. Group 1 included *F*. *ruetzleri* and *Gambierdiscus* ribotype 2 (Fig 2). Group 2 was *G*. *carpenteri*, *G*. *caribaeus* and *G*. *belizeanus* while Group 3 contained only *G*. *carolinianus*. It should be noted that while each of the species included in the preceding analysis exhibited low toxicity relative to *G*. *excentricus*, significant differences in toxicity were found among the lower toxicity species (Fig 2) (Table 1).

**Fig 3:**
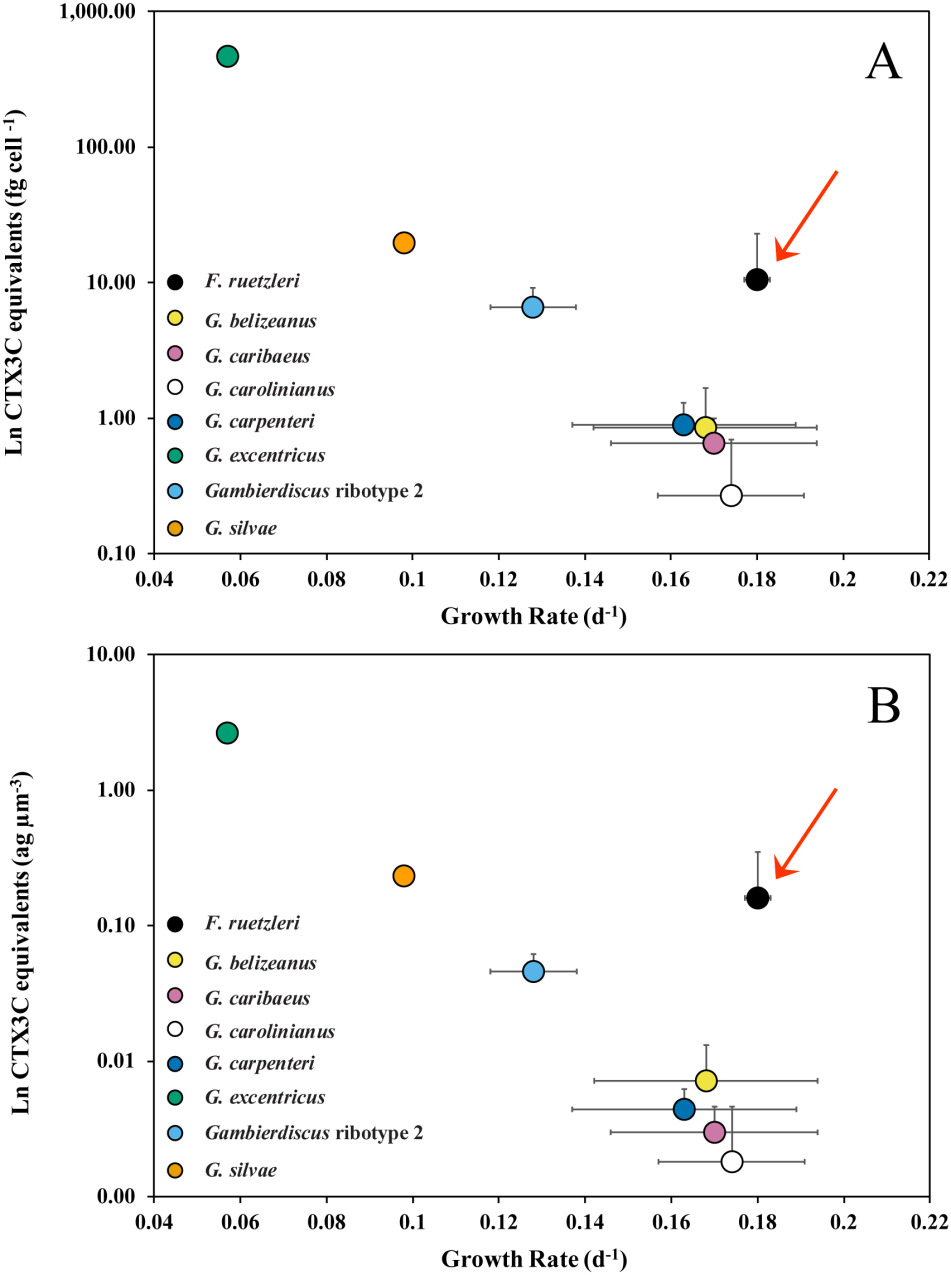
Ciguatoxicity versus growth rate. Natural log of cellular toxicity versus growth rate for each of the *Gambierdiscus* and *Fukuyoa* species normalized (A) to femtograms (fg) CTX3C eq. cell^-1^ and (B) attograms (ag) CTX3C eq. per μm^-3^ biovolume. Error bars = ± 1 standard deviation. The red arrows indicate data for *F*. *ruetzleri*, which had a higher toxicity than the *Gambierdiscus* species growing at a similar rate.

**Fig 4:**
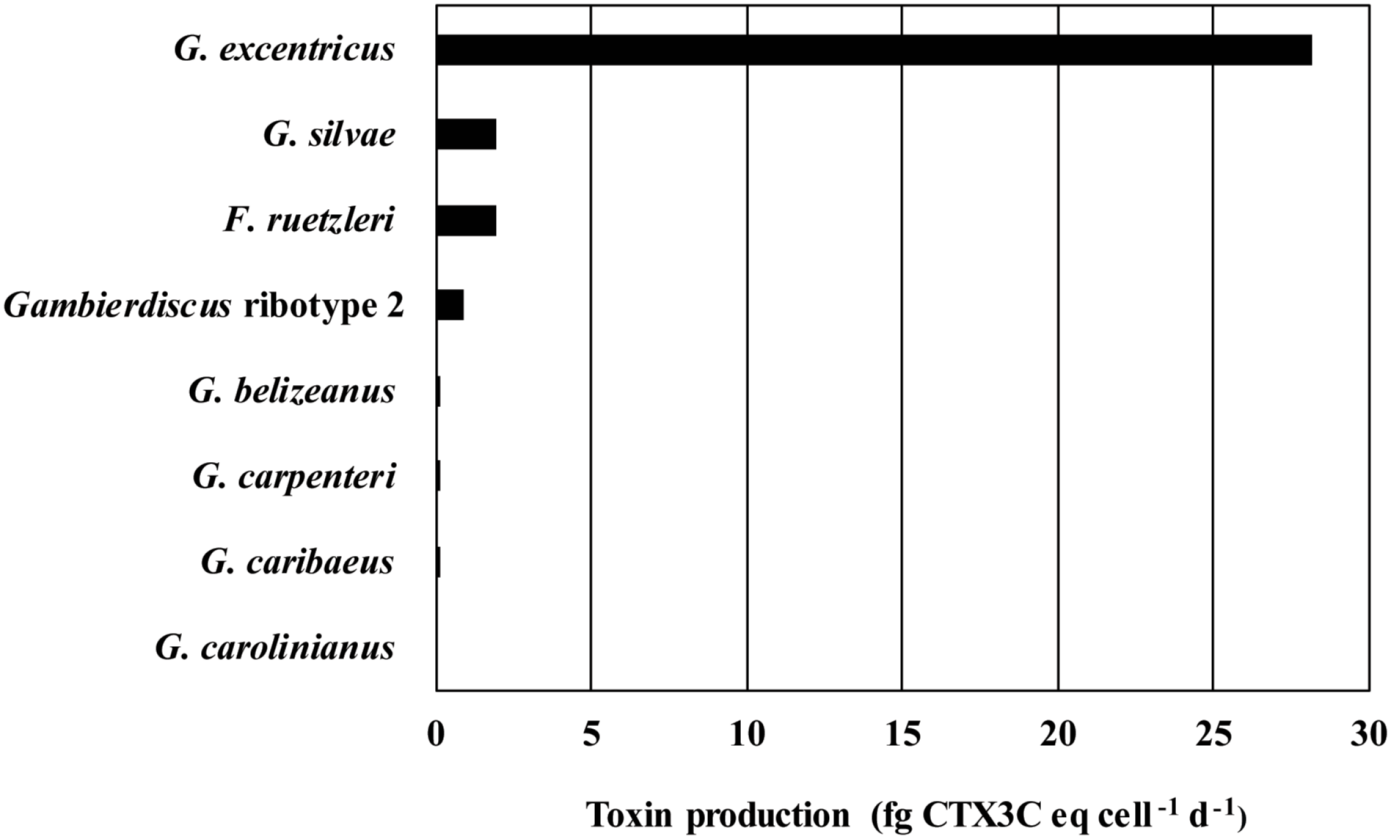
Toxin production rates. This figure shows the estimated toxin production (fg CTX3C eq. cell^-1^ d^-1^) rate for each species.

A plot of average *Gambierdiscus* growth rate versus average toxicity normalized on both a per cell and per biovolume basis showed the slower growing *Gambierdiscus* species were more toxic (Fig 3, S1 Fig). This increasing toxicity with declining growth rate followed an exponential relationship. Toxicity expressed as average toxin production rate fg CTX3C eq. cell^-1^ d^-1^ showed the same pattern of toxicity among species (Fig 4, S1 Fig). Based on the observed production rates, the difference between the most (*G*. *excentricus*) and least (*G*. *carolinianus*) toxic species was 613-fold. The equivalent difference between the most toxic and least toxic species based on toxicity per cell was ~1740-fold.

Only one *Fukuyoa* species was examined, so it was impossible to say if a similar relationship between growth rate and toxicity exists within this genus. It was apparent that toxicity for the *Fukuyoa* isolates tested was higher on a per cell and a per biomass basis compared to the *Gambierdiscus* species growing at a similar rate (Fig 3).

## Discussion

### Relative toxicity of *Gambierdiscus excentricus*

The *Gambierdiscus excentricus* isolate tested in this study was ~44- to >1,740-fold more toxic than the other species examined (~469 fg CTX3C eq. cell^-1^; Table 1, S3 Table). This result is consistent with the high toxicities reported for *G*. *excentricus* isolates from the Canary Islands (370–1,100 CTX1 eq. cell^-1^ and 1,425 CTX3C eq. cell^-1^; [38, 49]) and is similar to *G*. *polynesiensis*, the dominant toxin-producing species in the Pacific [29, 30]. To date, *G*. *polynesiensis* has not been identified from the eastern Atlantic, Caribbean or Gulf of Mexico (GOM), signifying that *G*. *excentricus* is the dominant CTX producer in the temperate and tropical regions of the eastern Atlantic, Caribbean and Gulf of Mexico (GOM) [8, 38, 49] (Table 1). In contrast, the range of toxicities exhibited by the other six *Gambierdiscus* and one *Fukuyoa* species examined varied from non-detectible to 24.5 fg CTX3C eq. cell^-1^.

The extent to which *G*. *excentricus* may dominate the CTX flux in Caribbean and GOM will depend on both its abundance and distribution. The tenant that environments fostering higher abundances of *G*. *excentricus* are more likely to produce ciguatoxic fish is put forward as a working hypothesis. Obtaining the data on abundance and distribution necessary to test this hypothesis will depend on quantitative species-specific molecular assays since *Gambierdiscus* species are not readily distinguished using light microscopy [31, 51]. Quantitative species-specific polymerase chain reaction (qPCR) assays are available for many Caribbean *Gambierdiscus* species, but not *G*. *excentricus* and the next most toxic species, *G*. *silvae*. Recently, PCR assays for *G*. *excentricus* and *G*. *silvae* were developed in our laboratory, but they have not yet been validated for quantitative (qPCR) estimation of cell abundances (unpublished). However, PCR screening on a limited number of field samples and newly isolated cultures allowed us to begin defining the geographic ranges of these species. *Gambierdiscus excentricus* was found in the Florida Keys, USA and the Bahamas, while *G*. *silvae* was present in the Bahamas, Saint Croix, and the U.S. Virgin Islands. Combining these data with those from the literature confirmed the minimum geographic range of *G*. *excentricus* extends from the northwest coast of Africa to southern Florida, USA and the southeast coast of Brazil [52, 53]. *Gambierdiscus silvae* ranges from the Canary Islands through the eastern and western Caribbean [8, 20]. More extensive sampling using species-specific qPCR assays has shown that *F*. *ruetzleri*, *G*. *belizeanus*, *G*. *caribaeus*, *G*. *carolinianus*, *G*. *carpenteri* and *Gambierdiscus* ribotype 2 are ubiquitously distributed throughout the Caribbean and GOM [8]. It is likely *G*. *excentricus* and *G*. *silvae* share an equally wide distribution. This suggests the contribution of *G*. *excentricus* to the overall toxin flux depends primarily on their relative abundance. The average toxin rate is 28.1 fg CTX3C eq. cell^-1^ d^-1^ for *G*. *excentricus* the most toxic species, 1.9 for the next most toxic species, *G*. *silvae* and *F*. *ruetzleri*, and 0.05 for *G*. *carolinianus*, the lest toxic species (Fig 3). If a population consisted of only *G*. *carolinianus* and *G*. *excentricus*, *G*. *excentricus* need only make up 0.16% of the total population to produce as much toxin as *G*. *carolinianus*. If the population contained only *G*. *silvae*, *F*. *ruetzleri* and *G*. *excentricus*, *G*. *excentricus* would have to make up 6.3% of the population on average to produce as many CTX equivalents as the other two species combined.

If *G*. *excentricus* is confirmed as the primary CTX producing species in the Atlantic, fully investigating its role in causing CFP may require careful chemical characterization of the specific CTX congeners it produces. That characterization would help facilitate development of LC-MS toxin-specific analytical methods capable of answering whether the low toxicity Atlantic *Gambierdiscus* and *Fukuyoa* species produce the same analogs in lesser quantities than *G*. *excentricus*, or only analogs of lower toxicity [26, 38, 49].

### Within species versus among species differences in CTX toxicity

A long-standing question in ciguatera research is the extent to which CFP risk is dependent on variations in toxicity among species versus between species [8]. Results of a Kruskal-Wallis nonparametric one factor ANOVA showed significant differences in CTX toxicity exist among the various *Gambierdiscus* species tested (Fig 2), confirming between species differences in toxicity are, on average, greater than among isolates of the same species (S3 Table). Though the within species variation for *G*. *excentricus* toxicity was not measured in this study, comparison with estimates in Fraga et al. [38] indicate within species variation is ~3-fold (370 to 1,100 fg P-CTX-1B eq. cell^-1^; n = 3). Other studies using CBA-N2a showed a similar within species variation in toxicity—0.6–2.7 fg CTX3C eq. cell^-1^ (n = 3) for *G*. *australes* [49], 0–19.9 fg P-CTX-1 eq. cell^-1^ (n = 4) for *G*. *balechii* [54], 2.6–6.0 fg P-CTX^-1^ eq. cell^-1^ (n = 4) for *Gambierdiscus* sp. type 4 [55] and 10.3–12.4 fg CTX3C eq. cell^-1^ (n = 2) for *G*. *silvae* [49] (S1 Table). Cumulatively, these data are consistent with CTX risk being primarily dependent on species composition.

### Relationship between growth rate and toxicity

Chinain [29] proposed that slower *Gambierdiscus* cell growth was associated with higher toxin content per cell. Indeed, *G*. *polynesiensis*, the slowest growing Pacific species tested to date, is by far the most toxic. The trend holds true for the *Gambierdiscus* species measured in this study with the slowest growing species, *G*. *excentricus* exhibiting the highest toxicity (Figs 3 and 4; Table 1). These data are consistent with an evolutionary tradeoff between an investment in growth versus the production of defensive compounds as observed in other harmful algal species [41, 56–58]. It is also noteworthy that relationship between toxicity and growth is exponential and not linear (Fig 3; [49]).

### Estimating CTX fluxes in the environment

Quantifying the contribution of various *Gambierdiscus* and *Fukuyoa* species to the flux of CTXs in the environment requires simultaneous determination of the species abundances and the amount of CTX being produced by each species. Undertaking such studies would be both expensive and technically challenging, especially since the full suite of species and the toxins they produce is unknown. A potentially, more tractable approach to understanding how different species may contribute to overall toxin fluxes is to incorporate the average toxicities into a physiologically-based *Gambierdiscus* growth rate model [15]. This approach would identify regions in the Caribbean and GOM where CTX fluxes may be highest. Model runs could also be adjusted to estimate how different relative abundances of low and high toxicity species would affect the magnitude of toxin flux. Explicit assumptions underlying this approach are that 1) average toxin concentrations represent the toxicity of the population as a whole and, 2) CFP risk is proportional to the toxicities of the *Gambierdiscus* and *Fukuyoa* species themselves.

The use of average toxicities in models is consistent with our knowledge of microalgal population genetics. Numerous studies have shown that algal populations maintain a high diversity of genotypes even during intense blooms, i.e. they are not dominated by only a few genotypes [59–64]. Averaging the toxicities of different isolates approximates population level toxicities. The relevance of using the toxicity of CTX-producing species to predict risk is supported by studies showing that as CTX congeners bioaccumulate in the food chain, some remain the same while others are biologically modified to have higher toxicities than their parent compounds [44, 65–67]. As a result, the toxicities remain the same or increase in the food chain so *Gambierdiscus* and *Fukuyoa* toxicities provide minimum estimates of CFP risk.

### Management implications

The results of this study have implications for managing CFP risk. Ideally, risk would be routinely assessed in an institutionalized surveillance system by quantitatively measuring a standard suite of CTXs in fish using LC-MS. Unfortunately, this is not practical because of the lack of certified standards and the high cost of analytical methods [47]. Until these obstacles are overcome, the problem requires a two-tiered approach. The first tier includes monitoring for increased cell abundances to determine elevated CFP risk and understanding the environmental conditions conducive to high *Gambierdiscus/Fukuyoa* abundance. The second tier includes the use of qPCR assays to determine the *Gambierdiscus/Fukuyoa* species composition with a focus on the relative numbers of *G*. *excentricus* in the Caribbean.

With respect to the first approach, it is known that CFP events can occur from one month to a year following a significant increase in *Gambierdiscus* cell densities [28, 68–70]. Consequently, genus-level cell counts using light microscopy [71] can provide first order estimates of CFP risk, but cannot predict severity. Despite this limitation, using this approach can provide managers an indication of when and where CFP risk may be elevated [72].

Interpretation of microscopic *Gambierdiscus* and *Fukuyoa* cell abundances can be further informed by understanding the environmental conditions that promote growth. Laboratory and field studies indicate temperature is the primary environmental factor regulating growth of *Gambierdiscus* and *Fukuyoa* species [15, 73, 74]. Modeling studies have also shown that in terms of broad patterns, annual temperature cycles can predict the regions where CFP risk is highest [75]. It is also known that CFP causing dinoflagellate species prefer habitats with low turbulence, appropriate substrate (macrophytes, algal turfs, coral rubble, seagrasses, etc.), nutrients supplied from the benthos or other sources, little or no direct runoff from land, and light levels >10 and < 200–700 μmol photons m^-2^ s^-1^ depending on species [8, 14, 18, 53, 71, 73, 74, 76–79]. The low light requirements of these species mean that habitats down to 50 meters or more may be capable of supporting substantial populations [79]. As GIS databases detailing habitat types throughout the Caribbean and Gulf of Mexico improve, they can be used in combination with the physiologically-based models to predict areas of higher CFP risk.

The second tier approach would use qPCR assays and focus on *G*. *excentricus* if it is confirmed as the dominant source of CTX in the Caribbean [23, 51]. Quantitative PCR assays are routinely used to monitor harmful algae in many regions of the world [80–83]. Only lack of resources keeps this from being possible throughout the Caribbean. Ultimately, as LC-MS methods become more cost effective, and high CFP risk areas are identified, the logical course is to use cell-based monitoring to focus on samples that need to be tested for toxins.

### Conclusions

*Gambierdiscus excentricus* was significantly more toxic than the other *Gambierdiscus* and the single species of *Fukuyoa* examined in this study from the Caribbean and GOM. Even with its slow growth rate, it is likely *G*. *excentricus* contributes disproportionally large fluxes of CTXs in the food chain. Overall, toxicity was inversely related to growth rate, indicating a tradeoff between investments of cellular resources in growth versus defensive compounds. Monitoring overall *Gambierdiscus* and *Fukuyoa* cell densities using genus-specific light microcopy may provide insight into when CFP risks are of concern, but cannot predict the severity of events. Despite this limitation, a cell-based approach can be used to predict first order risk assessment when no other method is available. If research confirms the hypothesis that one or a relatively few species produce most of the ciguatoxins entering the food web, then monitoring of those species using species-specific qPCR or other molecular assays will support more accurate assessments of CFP risk. Ecological models based on the physiological and ecological preferences of the key toxin producing species, also offer a way to cost effectively identify time periods and locations when CFP risk is the highest and when more expensive testing using LC-MS methods are warranted.

## Supporting information

S1 Table Comprehensive table showing what is known about CTX and MTX production by *Gambierdiscus* and *Fukuyoa* isolates not included in this study. (DOCX)

S2 Table Comparison of the *Gambierdiscus* and *Fukuyoa* growth rate estimates determined in this study versus rates published in other studies. (DOCX)

S3 Table A. Ratio of the highest toxicity isolate divided by the lowest isolate in fg CTX3C eq. cell^-1^. (DOCX)

S1 Fig Ciguatoxicity versus growth rate plotted on a linear scale. Cellular toxicity versus growth rate for each of the *Gambierdiscus* and *Fukuyoa* species normalized (A) to femtograms (fg) CTX3C eq. cell^-1^ and (B) attograms (ag) CTX3C eq. per μm^-3^ biovolume. Error bars = ± 1 standard deviation. This graph visually demonstrates the large difference in variation in toxicity of *G*. *excentricus* relative to the other species.
(TIF)
