# Supplementary material for: Ciguatoxicity of Gambierdiscus and Fukuyoa species from the Caribbean and Gulf of Mexico
Source: PLoS One. 2017 Oct 18;12(10):e0185776. doi: 10.1371/journal.pone.0185776 (PMC5646788; doi:10.1371/journal.pone.0185776)
Supplement: S1 Table — (DOCX) [file pone.0185776.s001.docx]

**Supporting information**

**S1 Table. Comprehensive table showing what is known about CTX and MTX production by *Gambierdiscus* and *Fukuyoa* isolates not included in this study.** + = either CTX or MTX detected, NT = not tested, - = no measurable toxicity (i.e. below detection limits for the specific method being used), "p-" detonates "putative" congener identification, and ? = extraction method may not have adequately separated CTX and MTX, further investigation is required to confirm presence of CTX or MTX. For the undescribed species a "+" in the CTX column = low toxicity (>2,000 cells per mouse unit), ++ = moderate toxicity (~500 to 2000 cells per mouse unit, +++ = high toxicity (<500 cells per mouse unit) as estimated in [1]. For the MTX study done using the hemolytic red blood cell assay + = EC_50_ >1000 cells, ++ = EC_50_ 100 to 1000 cells, +++= EC_50_ of 10 to 100 cells, [1]. Other studies with +, ++ and +++ indicate low, intermediate and high toxicities relative to other species measured using the same method. The data from Lewis et al. 2016 [63] indicates the potential number of CTX or MTX congeners present in algal extracts after fractionation and screening of each fraction using a sodium flux assay. In some studies, the presences of a putative CTX or MTX congener could be detected, but below concentrations that would allow accurate quantification. This situation is denoted by "-" to indicated below level for accurate quantification followed by the putative congener detected. NCMA = National Center for Marine Algae and Microbiota formally Center for Culture of Marine Phytoplankton (CCMA).

| **Species** | **Isolate** | **CTX** | **MTX** | **Location** | **Assay employed** | **Reference** |
| --- | --- | --- | --- | --- | --- | --- |
| ***F. ruetzleri*** | Gam 1 | NT | +++ | Southwater Cay, Belize | Hemolytic red blood cell assay | Holland et al. 2013  [1] |
|  | Gam 1 | +  3 | +  1 | Southwater Cay, Belize | Fluorescent calcium flux assay | Lewis et al. 2016  [2] |
|  | Gam 1 | NT | +  p-MTX3 | Southwater Cay, Belize | LC-LRMS/MS | Pisapia et al. 2017  [3] |
|  | NC Yasu | NT | + | North Carolina, continental shelf, USA | Hemolytic red blood cell assay | Holland et al. 2013  [1] |
|  | NOAA 3 | + | NT | South Water Cay, Belize | LC-MS/MS | Roeder et al. 2010  [4] |
|  | WH55 Gam4 | NT | +  p-MTX3 | Flower Garden Banks National Marine Sanctuary (West Bank),  Northwestern Gulf of Mexico, USA | LC-LRMS/MS | Pisapia et al. 2017  [3] |
|  |  |  |  |  |  |  |
| ***F. paulensis*** | Dn35EHU | + | + | Formentera Island, Western Mediterranean Sea | Mouse bioassay | Laza-Martinez et al. 2016  [5] |
|  |  |  |  |  |  |  |
| ***F. yasumotoi*** | Single isolated not designated | NT | + | Pulau Hantu Island, Singapore | Mouse bioassay | Holmes et al. 1998  [6] |
|  |  |  |  |  |  |  |
| ***F*. cf. *yasumotoi*** | CAWD210 | -  (pg CTX cell^-1^) | -  p-MTX3  (pg MTX cell^-1^) | Te Uenga Bay, Bay of Islands, Northland, New Zealand | LC–MS/MS | Rhodes et al. 2014  [7] |
|  | CAWD211 | -  (pg CTX cell^-1^) | -  p-MTX3  (pg MTX cell^-1^) | Te Uenga Bay, Bay of Islands, Northland, New Zealand | LC–MS/MS | Rhodes et al. 2014  [7] |
|  |  |  |  |  |  |  |
| ***G. australes*** | #2V | -  (pg CTX cell^-1^) | +  p-MTX-1  5.8  (pg MTX cell^-1^) | North Meyer Island, Kermadec Islands, Pacific | LC-MS/MS | Rhodes et al. 2016  [8] |
|  | #3–5 | -  (pg CTX cell^-1^) | +  pMTX-1  2.4  (pg MTX cell^-1^) | North Meyer Island, Kermadec Islands, Pacific | LC-MS/MS | Rhodes et al. 2016  [8] |
|  | #5a | -  (pg CTX cell^-1^) | +  p-MTX-1  3.6  (pg MTX cell^-1^) | North Meyer Island, Kermadec Islands, Pacific | LC-MS/MS | Rhodes et al. 2016  [8] |
|  | #5h | -  (pg CTX cell^-1^) | +  p-MTX-1  8.5  (pg MTX cell^-1^) | North Meyer Island, Kermadec Islands, Pacific | LC-MS/MS | Rhodes et al. 2016  [8] |
|  | CAWD149 | -  (pg CTX cell^-1^) | +  p-MTX3  8.3  (pg MTX cell^-1^) | Rarotonga, Cook Islands, Pacific Ocean | LC–MS/MS | Rhodes et al. 2014  [7] |
|  | CAWD216 | -  (pg CTX cell^-1^) | -  p-MTX3  (pg MTX cell^-1^) | Rarotonga, Cook Islands, Pacific Ocean | LC–MS/MS | Rhodes et al. 2014  [7] |
|  | CAWD244 | -  (pg CTX cell^-1^) | +  p-MTX-1  5.9  (pg MTX cell^-1^) | North Meyer Island, Kermadec Islands, Pacific | LC-MS/MS | Rhodes et al. 2016  [8] |
|  | CAWD245 | -  (pg CTX cell^-1^) | +  p-MTX-1  0.3  (pg MTX cell^-1^) | North Meyer Island, Kermadec Islands, Pacific | LC-MS/MS | Rhodes et al. 2016  [8] |
|  | CAWD246 | -  (pg CTX cell^-1^) | +  p-MTX-1  36.6  (pg MTX cell^-1^) | North Meyer Island, Kermadec Islands, Pacific | LC-MS/MS | Rhodes et al. 2016  [8] |
|  | CAWD248 | -  (pg CTX cell^-1^) | +  p-MTX-1  14.7  (pg MTX cell^-1^) | North Meyer Island, Kermadec Islands, Pacific | LC-MS/MS | Rhodes et al. 2016  [8] |
|  | CAWD254  Mac3-o | -  (pg CTX cell^-1^) | -  (pg MTX cell^-1^) | Macauley Island,  Kermadec Islands, Pacific | LC-MS/MS | Rhodes et al. 2017  [9] |
|  | CAWD255  Mac1-b | -  (pg CTX cell^-1^) | +  36  (pg MTX cell^-1^) | Macauley Island,  Kermadec Islands, Pacific | LC-MS/MS | Rhodes et al. 2017  [9] |
|  | CAWD256  Mac3-c | -  (pg CTX cell^-1^) | +  31  (pg MTX cell^-1^) | Macauley Island,  Kermadec Islands, Pacific | LC-MS/MS | Rhodes et al. 2017  [9] |
|  | CBaii | -  (pg CTX cell^-1^) | +  p-MTX-1  4.7  (pg MTX cell^-1^) | North Meyer Island, Kermadec Islands, Pacific | LC-MS/MS | Rhodes et al. 2016  [8] |
|  | CBc | -  (pg CTX cell^-1^) | +  p-MTX-1  17.7  (pg MTX cell^-1^) | North Meyer Island, Kermadec Islands, Pacific | LC-MS/MS | Rhodes et al. 2016  [8] |
|  | CBo | -  (pg CTX cell^-1^) | -  p-MTX-1  (pg MTX cell^-1^) | North Meyer Island, Kermadec Islands, Pacific | LC-MS/MS | Rhodes et al. 2016  [8] |
|  | CCMP 1653 | NT | +  p-MTX3 | Tern Island, Hawaii, USA | LC-LRMS/MS | Pisapia et al. 2017  [3] |
|  | CCMP 1653 | +  2.7 ± 0.6  (fg CTX3C eq. cell^-1^) | +  5.0 ± 0.5  (pg MTX eq. cell^−1^) | Tern Island, Hawaii, USA | N2a cytotoxicity assay | Pisapia et al. 2017  [10] |
|  | CCMP 1653 (NCMA) | + | NT | Tern Island, Hawaii, USA | LC-MS/MS | Roeder et al. 2010  [4] |
|  | HI9 gam | NT | + | Waikiki Beach, Hawaii, USA | Hemolytic red blood cell assay | Holland et al. 2013  [1] |
|  | Mac2-a | -  (pg CTX cell^-1^) | +  25  (pg MTX cell^-1^) | Macauley Island,  Kermadec Islands, Pacific | LC-MS/MS | Rhodes et al. 2017  [9] |
|  | Mac2-b | -  (pg CTX cell^-1^) | +  12  (pg MTX cell^-1^) | Macauley Island,  Kermadec Islands, Pacific | LC-MS/MS | Rhodes et al. 2017  [9] |
|  | Mac2-c | -  (pg CTX cell^-1^) | +  22  (pg MTX cell^-1^) | Macauley Island,  Kermadec Islands, Pacific | LC-MS/MS | Rhodes et al. 2017  [9] |
|  | Mac3-a | -  (pg CTX cell^-1^) | +  14  (pg MTX cell^-1^) | Macauley Island,  Kermadec Islands, Pacific | LC-MS/MS | Rhodes et al. 2017  [9] |
|  | Mac3-b | -  (pg CTX cell^-1^) | +  20  (pg MTX cell^-1^) | Macauley Island,  Kermadec Islands, Pacific | LC-MS/MS | Rhodes et al. 2017  [9] |
|  | Mac3-e | -  (pg CTX cell^-1^) | +  19  (pg MTX cell^-1^) | Macauley Island,  Kermadec Islands, Pacific | LC-MS/MS | Rhodes et al. 2017  [9] |
|  | Mac3-i | -  (pg CTX cell^-1^) - | +  3  (pg MTX cell^-1^) | Macauley Island,  Kermadec Islands, Pacific | LC-MS/MS | Rhodes et al. 2017  [9] |
|  | Mac3-m | -  (pg CTX cell^-1^) | +  19  (pg MTX cell^-1^) | Macauley Island,  Kermadec Islands, Pacific | LC-MS/MS | Rhodes et al. 2017  [9] |
|  | Mac3-n | -  (pg CTX cell^-1^) | +  5  (pg MTX cell^-1^) | Macauley Island,  Kermadec Islands, Pacific | LC-MS/MS | Rhodes et al. 2017  [9] |
|  | Mac4-e | -  (pg CTX cell^-1^) | +  10  (pg MTX cell^-1^) | Macauley Island,  Kermadec Islands, Pacific | LC-MS/MS | Rhodes et al. 2017  [9] |
|  | Mac4-f | -  (pg CTX cell^-1^) | +  9  (pg MTX cell^-1^) | Macauley Island,  Kermadec Islands, Pacific | LC-MS/MS | Rhodes et al. 2017  [9] |
|  | Mac4-fuk | -  (pg CTX cell^-1^) | +  21  (pg MTX cell^-1^) | Macauley Island,  Kermadec Islands, Pacific | LC-MS/MS | Rhodes et al. 2017  [9] |
|  | Mac4-g | -  (pg CTX cell^-1^) | +  27  (pg MTX cell^-1^) | Macauley Island,  Kermadec Islands, Pacific | LC-MS/MS | Rhodes et al. 2017  [9] |
|  | Mac5-a | -  (pg CTX cell^-1^) | +  16  (pg MTX cell^-1^) | Macauley Island,  Kermadec Islands, Pacific | LC-MS/MS | Rhodes et al. 2017  [9] |
|  | Mac5-b | -  (pg CTX cell^-1^) | +  15  (pg MTX cell^-1^) | Macauley Island,  Kermadec Islands, Pacific | LC-MS/MS | Rhodes et al. 2017  [9] |
|  | Mac5-c | -  (pg CTX cell^-1^) | +  8  (pg MTX cell^-1^) | Macauley Island,  Kermadec Islands, Pacific | LC-MS/MS | Rhodes et al. 2017  [9] |
|  | Mac5-e | -  (pg CTX cell^-1^) | +  20  (pg MTX cell^-1^) | Macauley Island,  Kermadec Islands, Pacific | LC-MS/MS | Rhodes et al. 2017  [9] |
|  | Mac5-f | -  (pg CTX cell^-1^) | +  8  (pg MTX cell^-1^) | Macauley Island,  Kermadec Islands, Pacific | LC-MS/MS | Rhodes et al. 2017  [9] |
|  | Mac5-g | -  (pg CTX cell^-1^) | +  7  (pg MTX cell^-1^) | Macauley Island,  Kermadec Islands, Pacific | LC-MS/MS | Rhodes et al. 2017  [9] |
|  | Mac5-j | -  (pg CTX cell^-1^) | +  32  (pg MTX cell^-1^) | Macauley Island,  Kermadec Islands, Pacific | LC-MS/MS | Rhodes et al. 2017  [9] |
|  | Mac5-l | -  (pg CTX cell^-1^) | +  6  (pg MTX cell^-1^) | Macauley Island,  Kermadec Islands, Pacific | LC-MS/MS | Rhodes et al. 2017  [9] |
|  | Mac65-a | -  (pg CTX cell^-1^) | +  18  (pg MTX cell^-1^) | Macauley Island,  Kermadec Islands, Pacific | LC-MS/MS | Rhodes et al. 2017  [9] |
|  | MG-4 | -  ­(fg CTX3C eq. cell^-1^) | NT | Rikitea, Mangareva | Receptor binding assay | Chinain et al 2010  [11] |
|  | MUR-6 | 17  (fg CTX3C eq. cell^-1^) | NT | Moruroa, French Polynesia | Receptor binding assay | Chinain et al 2010  [11] |
|  | MUR-14 | 30  (fg CTX3C eq. cell^-1^) | NT | Moruroa, French Polynesia, | Receptor binding assay | Chinain et al 2010  [11] |
|  | RAI-5 | -  (fg CTX3C eq. cell^-1^) | NT | Motu Femme, Raivavae, French Polynesia | Receptor binding assay | Chinain et al 2010  [11] |
|  | RAV-92 | +  4  (×10^−4^ MU 1,000 cells^−1^) | +  0.2  (MU 1,000 cells^−1^) | Rairua, Raivavae Island, Australes Archipelago, French Polynesia | Mouse bioassay | [Chinain et al. 1999](#_bookmark33)  [12] |
|  | RAV-92 | 20  (fg CTX3C eq. cell^-1^) | NT | Rairua, Raivavae Island, Australes Archipelago, French Polynesia | Receptor binding assay | Chinain et al 2010  [11] |
|  | TB-1 | 22  (fg CTX3C eq. cell^-1^) | NT | Mataura, Tubuai, French Polynesia | Receptor binding assay | Chinain et al 2010  [11] |
|  | S080911_1 | +  670  (×10^−4^ MU 1,000 cells^−1^) | +  67  (×10^−4^ MU 1,000 cells^−1^) | Kutsu, Susaki, Kochi, Japan | Mouse bioassay | Nishimura et al. 2013  [13] |
|  | S080911_1 | NT | +  p-MTX3  22.6 ± 0.5  (pg MTX eq. cell^−1^) | Kutsu, Susaki, Kochi, Japan | LC-LRMS/MS | Pisapia et al. 2017  [3] |
|  | VGO1178 | NT | +  p-MTX3 | Punta Hidalgo, Tenerife, Canary Islands | LC-LRMS/MS | Pisapia et al. 2017  [3] |
|  | VGO1178 | +  1.4 ± 0.4  (fg CTX3C eq. cell^-1^) | +  4.3 ± 1.2  (pg MTX eq. cell^−1^) | Punta Hidalgo, Tenerife, Canary Islands, Spain | N2a cytotoxicity assay | Pisapia et al. 2017  [10] |
|  | VGO1181 | NT | +  p-MTX3 | Punta Hidalgo, Tenerife, Canary Islands, Spain | LC-LRMS/MS | Pisapia et al. 2017  [3] |
|  | VGO1181 | +  0.6 ± 0.3  (fg CTX3C eq. cell^-1^) | +  4.7 ± 2.8  (pg MTX eq. cell^−1^) | Punta Hidalgo, Tenerife, Canary Islands, Spain | N2a cytotoxicity assay | Pisapia et al. 2017  [10] |
|  | W B Gam 3 | - | +  2 | Waikiki Beach, Hawaii, USA | Fluorescent calcium flux assay | Lewis et al. 2016  [2] |
|  |  |  |  |  |  |  |
| ***G. balechii*** (*Gambierdiscus* sp. type 6) | 1012M4D02 | +  0.14 ± 0.01  (fg P-CTX-1 eq cell^-1^) | NT | Marakei Island, Kiribati, Pacific | N2a cytotoxicity assay | Dai et al. 2017  [14] |
|  |  |  |  |  |  |  |
|  | 1112M1D08 | +  1.13 ± 0.17  (fg P-CTX-1 eq cell^-1^) | NT | Marakei Island, Kiribati, Pacific Marakei Island, Kiribati, Pacific | N2a cytotoxicity assay | Dai et al. 2017  [14] |
|  | 1112M1D09 1 | +  1.11 ± 0.19  (fg P-CTX-1 eq cell^-1^) | NT | Marakei Island, Kiribati, Pacific | N2a cytotoxicity assay | Dai et al. 2017  [14] |
|  | 1112M1M03 | +  19.9 ± 9.55  (fg P-CTX-1 eq cell^-1^) | NT | Marakei Island, Kiribati, Pacific | N2a cytotoxicity assay | Dai et al. 2017  [14] |
|  | T6PrGd03N | -  (fg P-CTX-1 eq cell^-1^) | NT | Rawa Island, Malaysia | N2a cytotoxicity assay | Dai et al. 2017  [14] |
|  | VGO917 | + | + | Manado, Celebes Seat, Pacific Ocean | Mouse bioassay | Fraga et al. 2016  [15] |
|  | VGO917 | NT | +  p-MTX3 | Manado, Celebes Seat, Pacific Ocean | LC-LRMS/MS | Pisapia et al. 2017  [3] |
|  | VGO917 | +  3.4 ± 1.5  (fg CTX3C eq. cell^-1^) | +  19.9 ± 2.9  (pg MTX eq. cell^−1^) | Manado, Celebes Seat, Pacific Ocean | N2a cytotoxicity assay | Pisapia et al. 2017  [10] |
|  | VGO920 | NT | +  p-MTX3 | Manado, Celebes Seat, Pacific Ocean | LC-LRMS/MS | Pisapia et al. 2017  [3] |
|  |  |  |  |  |  |  |
| ***G. belizeanus*** | CCMP 399 | NT | ++ | St. Barthelemy, Collectivity of France, Caribbean | Hemolytic red blood cell assay | Holland et al. 2013  [1] |
|  | CCMP 399 | +  4 | +  4 | St. Barthelemy, Collectivity of France, Caribbean | Fluorescent calcium flux assay | Lewis et al. 2016  [2] |
|  | CCMP 399 | NT | +  p-MTX3 | St. Barthelemy, Collectivity of France, Caribbean | LC-LRMS/MS | Pisapia et al. 2017  [3] |
|  | CCMP 401 (NCMA) | + | NT | St. Barthelemy, Collectivity of France, Caribbean | LC-MS/MS | Roeder et al. 2010  [4] |
|  | Keys Gam 1 | NT | ++ | Florida Keys, USA | Hemolytic red blood cell assay | Holland et al. 2013  [1] |
|  | Keys Gam 1 | NT | +  p-MTX3 | Florida Keys, USA | LC-LRMS/MS | Pisapia et al. 2017  [3] |
|  | STB-1 | +  123  (fg CTX3C eq. cell^-1^) | NT | St Barthélémy, Caribbean | Receptor binding assay | Chinain et al 2010  [11] |
|  | ST1 Gam F4 | NT | + | St. Thomas, US Virgin Islands, Caribbean | Hemolytic red blood cell assay | Holland et al. 2013  [1] |
|  | ST1 Gam F4 | NT | +  p-MTX3 | St. Thomas, US Virgin Islands, Caribbean | LC-LRMS/MS | Pisapia et al. 2017  [3] |
|  |  |  |  |  |  |  |
| ***G. caribaeus*** | Algae 1 gam 1 | NT | ++ | Cancun, Mexico | Hemolytic red blood cell assay | Holland et al. 2013  [1] |
|  | Jamaica 1 gam1 | NT | + | Ocho Rios, Jamaica | Hemolytic red blood cell assay | Holland et al. 2013  [1] |
|  | Bill Hi Gam8 | NT | +  p-MTX2, 3 | Waikiki Beach, Honolulu, Hawaii, USA | LC-LRMS/MS | Pisapia et al. 2017  [3] |
|  | Bill Hi Gam | +  1.6 ± 1.0  (fg CTX3C eq. cell^-1^) | +  5.3 ± 1.0  (pg MTX eq. cell^−1^) | Waikiki Beach, Honolulu, Hawaii, USA | N2a cytotoxicity assay | Pisapia et al. 2017  [10] |
|  | BB Gam 4 | NT | ++ | Bathtub Beach, Florida , USA | Hemolytic red blood cell assay | Holland et al. 2013  [1] |
|  | BRP Gam 4 | NT | ++ | Jupiter, Florida, USA | Hemolytic red blood cell assay | Holland et al. 2013  [1] |
|  | CBC Gam1 | NT | ++ | Carrie Bow Cay, Belize | Hemolytic red blood cell assay | Holland et al. 2013  [1] |
|  | CCMP 1651 (NCMA) | NT | + | Grand Cayman Island, Caribbean | Hemolytic red blood cell assay | Holland et al. 2013  [1] |
|  | CCMP 1651 (NCMA) | + | NT | Grand Cayman Island, Caribbean | LC-MS/MS | Roeder et al. 2010  [4] |
|  | CCMP 1651 (NCMA) | +  30.7 to 54.3  (fg C-CTX1-Eq. cell^-1^) | NT | Grand Cayman Island, Caribbean | N2A cytotoxicity assay | Lartigue et al 2009  [16] |
|  | CCMP 1651 (NCMA) | NT | +  p-MTX2,3 | Grand Cayman Island, Caribbean | LC-LRMS/MS | Pisapia et al. 2017  [3] |
|  | CCMP 1652 (NCMA) | + | NT | Mataiva, Tahiti, French Polynesia | LC-MS/MS | Roeder et al. 2010  [4] |
|  | CCMP 1733 (NCMA) | NT | ++ | Carrie Bow Cay, Belize | Hemolytic red blood cell assay | Holland et al. 2013  [1] |
|  | CCMP 1733 (NCMA) | + | NT | Carrie Bow Cay, Belize | LC-MS/MS | Roeder et al. 2010  [4] |
|  | CCMP 1733 (NCMA) | NT | +  p-MTX2,3 | Carrie Bow Cay, Belize | LC-LRMS/MS | Pisapia et al. 2017  [3] |
|  | Coral cove Gam 1 | NT | ++ | Jupiter, Florida, USA | Hemolytic red blood cell assay | Holland et al. 2013  [1] |
|  | Dive 1 fa Gam 1 | NT | ++ | Ft. Pierce, Florida, USA | Hemolytic red blood cell assay | Holland et al. 2013  [1] |
|  | Dive 1 fa Gam 1 | NT | +  p-MTX2,3 | Ft. Pierce, Florida, USA | LC-LRMS/MS | Pisapia et al. 2017  [3] |
|  | ETB Gam 6 | NT | ++ | Dry Tortugas, Gulf of Mexico | Hemolytic red blood cell assay | Holland et al. 2013  [1] |
|  | Gam 4 | NT | ++ | Belize | Hemolytic red blood cell assay | Holland et al. 2013 [1] |
|  | Gam19 | NT | + | Belize | Hemolytic red blood cell assay | Holland et al. 2013  [1] |
|  | Gam 19 | - | ++  3 | Carrie Bow Cay, Belize | Fluorescent calcium flux assay | Lewis et al. 2016  [2] |
|  | Keys Jar 7 Gam 7 | NT | ++ | Long Key, Florida Keys, USA | Hemolytic red blood cell assay | Holland et al. 2013  [1] |
|  | Jamaica Algae 1 Gam 1 | NT | ++ | Belize | Hemolytic red blood cell assay | Holland et al. 2013  [1] |
|  | Jar 12 Tow 3 | NT | ++ | Belize | Hemolytic red blood cell assay | Holland et al. 2013  [1] |
|  | Jar 17 gam | NT | + | Belize | Hemolytic red blood cell assay | Holland et al. 2013  [1] |
|  | Mexico Algae1 Gam1 | NT | ++  p-MTX2,3 | Mexico, western Caribbean | LC-LRMS/MS | Pisapia et al. 2017  [3] |
|  | Norval Cay | NT | ++ | Norval Cay, Belize | Hemolytic red blood cell assay | Holland et al. 2013  [1] |
|  | Outfish 7-1 | NT | ++ | Dry Tortugas, Gulf of Mexico | Hemolytic red blood cell assay | Holland et al. 2013  [1] |
|  | Outfish 7-3 | NT | ++ | Dry Tortugas, Gulf of Mexico | Hemolytic red blood cell assay | Holland et al. 2013  [1] |
|  | Pat Jar 2 Gam | NT | + | Oahu, Hawaii, USA | Hemolytic red blood cell assay | Holland et al. 2013  [1] |
|  | Pat HI Jar 2 Gam 2 | - | +  3 | Big Island, Hawaii, USA | Fluorescent calcium flux assay | Lewis et al. 2016  [2] |
|  | SW Gam 4 | NT | ++ | South Water Cay, Belize | Hemolytic red blood cell assay | Holland et al. 2013  [1] |
|  | SW Gam 5 | NT | ++ | South Water Cay, Belize | Hemolytic red blood cell assay | Holland et al. 2013  [1] |
|  | SJ 3 D7 | NT | ++ | St. John, US Virgin Islands, Caribbean | Hemolytic red blood cell assay | Holland et al. 2013  [1] |
|  | ST 1 C5 | NT | ++ | St. Thomas, US Virgin Islands, Caribbean | Hemolytic red blood cell assay | Holland et al. 2013  [1] |
|  | TC tow Gam 3 | NT | ++ | Twin Cays, Belize | Hemolytic red blood cell assay | Holland et al. 2013  [1] |
|  | WBHR 21 gam 2 | NT | + | Flower Garden Banks National Marine Sanctuary, Gulf of Mexico, USA | Hemolytic red blood cell assay | Holland et al. 2013  [1] |
|  | WBHR 26 gam | NT | + | Flower Garden Banks National Marine Sanctuary, Gulf of Mexico, USA | Hemolytic red blood cell assay | Holland et al. 2013  [1] |
|  |  |  |  |  |  |  |
| ***G. carolinianus*** | Algae Gam 1 | NT | + | Ocho Rios Jamaica | Hemolytic red blood cell assay | Holland et al. 2013  [1] |
|  | Big Fish Gam | NT | + | North Carolina, USA | Hemolytic red blood cell assay | Holland et al. 2013  [1] |
|  | Dive 1 Gam1 | NT | ++ | Carrie Bow Cay, Belize | Hemolytic red blood cell assay | Holland et al. 2013  [1] |
|  | Dive 1 Gam 1 | +  2 | +  2 | Carrie Bow Cay, Belize | Fluorescent calcium flux assay | Lewis et al. 2016  [2] |
|  | Elbow Cay | NT | ++ | Elbow Cay, Belize | Hemolytic red blood cell assay | Holland et al. 2013  [1] |
|  | ETB Exp 28 Gam 10 | NT | + | Dry Tortugas, Gulf of Mexico | Hemolytic red blood cell assay | Holland et al. 2013  [1] |
|  | ETB Exp 28 Gam 10 | NT | +  p-MTX3 | Dry Tortugas, Gulf of Mexico | LC-LRMS/MS | Pisapia et al. 2017  [3] |
|  | Gam 3 | NT | + | Crete, Greece | Hemolytic red blood cell assay | Holland et al. 2013  [1] |
|  | Greece Gam2 | NT | +  p-MTX3 | Crete, Greece | LC-LRMS/MS | Pisapia et al. 2017  [3] |
|  | Greece Gam2 | +  3.3 ± 0.6  (fg CTX3C eq. cell^-1^) | +  10.6 ± 0.4  (pg MTX eq. cell^−1^) | Crete, Greece | N2a cytotoxicity assay | Pisapia et al. 2017  [10] |
|  | Jamaica Algae Gam 1 | NT | ++ | Jupiter, Florida, USA | Hemolytic red blood cell assay | Holland et al. 2013  [1] |
|  | Jupiter Algae gam | NT | + | Jupiter, Florida, USA | Hemolytic red blood cell assay | Holland et al. 2013  [1] |
|  | Ken 3 | NT | + | Offshore North Carolina , USA | Hemolytic red blood cell assay | Holland et al. 2013  [1] |
|  | Kenny 6 | NT | + | Offshore North Carolina, USA | Hemolytic red blood cell assay | Holland et al. 2013  [1] |
|  | Kenny 6 | - | +  3 | Offshore North Carolina, USA | Fluorescent calcium flux assay | Lewis et al. 2016  [2] |
|  | Lob Rock N7 | NT | + | Offshore North Carolina, USA | Hemolytic red blood cell assay | Holland et al. 2013  [1] |
|  | Lob Rock N3 | NT | + | Offshore North Carolina, USA | Hemolytic red blood cell assay | Holland et al. 2013  [1] |
|  | Mex Algae Gam 1 | NT | ++ | Mexico | Hemolytic red blood cell assay | Holland et al. 2013  [1] |
|  | Pat HI Jar 3 Gam 9 | - | +  3 | Big Island, Hawaii, USA | Fluorescent calcium flux assay | Lewis et al. 2016  [2] |
|  | PRG Gam 1 | NT | + | Puerto Rico, USA | Hemolytic red blood cell assay | Holland et al. 2013  [1] |
|  | RROV5 | NT | + | Puerto Rico, USA | Hemolytic red blood cell assay | Holland et al. 2013  [1] |
|  | RROV5 | NT | +  p-MTX3 | Puerto Rico, USA | LC-LRMS/MS | Pisapia et al. 2017  [3] |
|  | ST1 F7 | NT | + | St. Thomas, US Virgin Islands, USA | Hemolytic red blood cell assay | Holland et al. 2013  [1] |
|  | WBHR 21 Gam | NT | ++ | Flower Garden Banks National Marine Sanctuary, Gulf of Mexico | Hemolytic red blood cell assay | Holland et al. 2013  [1] |
|  |  |  |  |  |  |  |
| ***G. carpenteri*** | Algae 2 gam 1 | NT | + | Ocho Rios, Jamaica | Hemolytic red blood cell assay | Holland et al. 2013  [1] |
|  | CCMP 1654 (NCMA) | NT | ++ | Guam, North Pacific, USA | Hemolytic red blood cell assay | Holland et al. 2013  [1] |
|  | CCMP 1654 (NCMA) | + | NT | Guam, North Pacific, USA | LC-MS/MS | Roeder et al. 2010  [4] |
|  | ETB Exp 24 Gam 1 | NT | + | Dry Tortugas, Gulf of Mexico | Hemolytic red blood cell assay | Holland et al. 2013  [1] |
|  | GT4 | NT | ++ | Carrie Bow Cay, Belize | Hemolytic red blood cell assay | Holland et al. 2013  [1] |
|  | GT4 | - | +  3 | Carrie Bow Cay, Belize | Fluorescent calcium flux assay | Lewis et al. 2016  [2] |
|  | GT4 | NT | +  p-MTX3 | Carrie Bow Cay, Belize | LC-LRMS/MS | Pisapia et al. 2017  [3] |
|  | Jamaica Algae2 Gam1 | NT | +  p-MTX3 | Ocho Rios, Jamaica | Hemolytic red blood cell assay | Holland et al. 2013  [1] |
|  | Jamaica Algae2 Gam1 | NT | +  p-MTX3 | Ocho Rios, Jamaica | LC-LRMS/MS | Pisapia et al. 2017  [3] |
|  | NOAA 12 | + | NT | South Water Cay, Belize | LC-MS/MS | Roeder et al. 2010  [4] |
|  | Pat HI jar 5 Gam 3 | NT | + | Oahu, Hawaii, USA | Hemolytic red blood cell assay | Holland et al. 2013  [1] |
|  | Pat HI Jar 7 Gam 11 | NT | +  3 | Waikiki Beach, Honolulu, Hawaii, USA | Fluorescent calcium flux assay | Lewis et al. 2016  [2] |
|  | Pat HI Jar 7 Gam 11 | NT | +  p-MTX3 | Waikiki Beach, Honolulu, Hawaii, USA | LC-LRMS/MS | Pisapia et al. 2017  [3] |
|  | Pat HI Jar 7 Gam 11 | +  1.4 ± 0.6  (fg CTX3C eq. cell^-1^) | +  6.3 ± 1.9  (pg MTX eq. cell^−1^) | Waikiki Beach, Honolulu, Hawaii, USA | N2a cytotoxicity assay | Pisapia et al. 2017  [10] |
|  | WBHR 21 | NT | + | Flower Garden Banks National Marine Sanctuary, Gulf of Mexico, USA | Hemolytic red blood cell assay | Holland et al. 2013  [1] |
|  | WBHR 21 | NT | +  p-MTX3 | Flower Garden Banks National Marine Sanctuary, Gulf of Mexico | LC-LRMS/MS | Pisapia et al. 2017  [3] |
|  |  |  |  |  |  |  |
| ***G. cheloniae*** | CAWD232 | - | -  p-MTX3  0.320  (0.221–0.426)  (mg/kg) | Highland Paradise, Rarotonga, Cook Islands | Mouse bioassay, LD50 by intraperitoneal injection, | Smith et al 2016  [17] |
|  | CAWD236 | - | -  p-MTX3  1.58  (1.11–2.09) (mg/kg) | Papua Passage, Rarotonga, Cook Islands | Mouse bioassay, LD50 by intraperitoneal injection | Smith et al 2016  [17] |
|  |  |  |  |  |  |  |
| ***G. excentricus*** | Pulley Ridge Gam 2 | NT | +  MTX4  22.9  (pg MTX eq. cell^−1^) | Southern Gulf of Mexico | LC-LRMS/MS | Pisapia et al. 2017  [3] |
|  | UNR-07 | NT | ++  MTX4  16.0 ± 2.3  (pg MTX eq. cell^−1^) | Armação dos Búzios, Rio de Janeiro, Brazil | LC-LRMS/MS | Pisapia et al. 2017  [3] |
|  | UNR-08 | NT | ++  MTX4  19.8 ± 6.4  (pg MTX eq. cell^−1^) | Armação dos Búzios, Rio de Janeiro, Brazil | LC-LRMS/MS | Pisapia et al. 2017  [3] |
|  | VGO790 | +++  1.10 ± 0.19  (pg CTX 1B eq. (cell^-1^) | +++  1.38 ± 0.31  (ng MTX eq. cell-^1^) | Punta Hidalgo, Tenerife, Canary Islands, Spain | N2a cytotoxicity assay | Fraga et al. 2011  [18] |
|  | VGO790 | NT | ++  MTX4  23.2  (pg MTX eq. cell^−1^) | Punta Hidalgo, Tenerife, Canary Islands, Spain | LC-LRMS/MS | Pisapia et al. 2017  [3] |
|  | VGO791 | +++  1.05 ± 0.18  (pg CTX 1B eq. cell^-1^) | +++  0.60 ± 0.24  (ng MTX eq. cell-^1^) | Punta Hidalgo, Tenerife, Canary Islands, Spain | CBA-N2a cytotoxicity assay | Fraga et al. 2011  [18] |
|  | VGO791 | NT | +++  MTX4  72.8 ± 8.5  (pg MTX eq. cell^−1^) | Punta Hidalgo, Tenerife, Canary Islands, Spain | LC-LRMS/MS | Pisapia et al. 2017  [3] |
|  | VGO791 | +++  1426 ± 55  (fg CTX3C eq. cell^-1^) | +++  85.7 ± 41.5  (pg MTX eq. cell^−1^) | Punta Hidalgo, Tenerife, Canary Islands, Spain | N2a cytotoxicity assay | Pisapia et al. 2017  [10] |
|  | VGO792 | +++  0.37 ± 0.17  (pg CTX1B eq. (cell^-1^) | +++  0.48 ± 0.16  (ng MTX eq. cell^-1^) | Punta Hidalgo, Tenerife, Canary Islands, Spain | CBA-N2a cytotoxicity assay | Fraga et al. 2011  [18] |
|  | VGO792 | NT | ++  MTX4  20.0 ± 2.9  (pg MTX eq. cell^−1^) | Punta Hidalgo, Tenerife, Canary Islands, Spain | LC-LRMS/MS | Pisapia et al. 2017  [3] |
|  | VGO1035 | NT | ++  MTX4  13  (pg MTX eq. cell^−1^) | Playa Las Cabras, La Palma, Canary Islands, Spain | LC-LRMS/MS | Pisapia et al. 2017  [3] |
|  |  |  |  |  |  |  |
| ***G. honu*** | CAWD233 | - | +  p-MTX3  0.20  (0.15–0.24)  (mg kg^-1^) | Betela, Cook Islands, Pacific | Mouse bioassay, LD_50_ by intraperitoneal injection | Rhodes et al 2017  [19] |
|  | CAWD242 | -  (pg CTX cell^-1^) | -  p-MTX3  (pg MTX cell^-1^) | North Meyer Island, Kermadec Islands, Pacific | LC-MS/MS | Rhodes et al. 2016  [8] |
|  | CAWD242 | -  (pg CTX cell^-1^) | -  p-MTX3  (pg MTX cell^-1^)  0.20  (0.07–0.23)  (mg kg^-1^) | North Meyer Island, Kermadec Islands, Pacific | Mouse bioassay, LD_50_ by intraperitoneal injection | Rhodes et al. 2017  [19] |
|  |  |  |  |  |  |  |
| ***G. lapillus*** | HG4 | ?  150  (mg kg-1) | + | Heron Island, Great Barrier Reef, Australia | Mouse bioassay, median lethal doses by intraperitoneal injection | Kretzschmar et al. 2017 [20] |
|  | HG6 | ?  0.78  (0.40 - 1.60)  (mg kg^-1^) | + | Heron Island, Great Barrier Reef, Australia | Mouse bioassay, median lethal doses by intraperitoneal injection | Kretzschmar et al. 2017  [20] |
|  | HG7 | ?  12.5  (10.1 - 15.3)  (mg kg^-1^) | + | Heron Island, Great Barrier Reef, Australia | Mouse bioassay, median lethal doses by intraperitoneal injection | Kretzschmar et al. 2017  [20] |
|  |  |  |  |  |  |  |
| ***G. pacificus*** | 3S0509-27 | +  0.011  (fg P-CTX-1 eq. cell^-1^) | NT | Marakei, Republic of Kiribati, Pacific ocean | CBA-N2a cytotoxicity assay | Xu et al. 2014  [21] |
|  | CAWD213 | -  (pg CTX cell^-1^) | -  p-MTX3  (pg CTX cell^-1^) | Rarotonga Lagoon, Cook Islands, Pacific | LC–MS/MS | Rhodes et al. 2014  [7] |
|  | CCMP 1650 (NCMA) | NT | + | Moorea, Society Islands, Pacific Ocean | Hemolytic red blood cell assay | Holland et al. 2013  [1] |
|  | CCMP 1650 (NCMA) | + | NT | Moorea, Society Islands, Pacific Ocean | LC-MS/MS | Roeder et al. 2010  [4] |
|  | CCMP 1650 (NCMA) | NT | +  p-MTX2,3 | Moorea, Society Islands, Pacific Ocean | LC-LRMS/MS | Pisapia et al. 2017  [3] |
|  | CCMP 1650 (NCMA) | +  12.1 ± 0.0  (fg CTX3C eq. cell^-1^) | ++  20.1 ± 5.9  (pg MTX eq. cell^−1^) | Moorea, Society Islands, Pacific Ocean | N2a cytotoxicity assay | Pisapia et al. 2017  [10] |
|  | G10DC | +  Tentatively  2,3-dihydroxyCTX3C,  51-hydroxyCTX3C  M-/L-*seco*-CTX3C | + | Malaysia | CBA-N2a cytotoxicity assay  LC-MS/MS | Caillaud et al. 2011  [22] |
|  | ET-2 | -  (fg CTX3C eq. cell^-1^) | NT | Mahu, Tubuai, French Polynesia | Receptor binding assay | Chinain et al 2010  [11] |
|  | G10DC | NT | +  p-MTX2,3 | Malaysia | LC-LRMS/MS | Pisapia et al. 2017  [3] |
|  | HO-91 | +  9  (×10^−4^ MU 1,000 cells^−1^) | +  0.7  (×10^−4^ MU 1,000 cells^−1^) | Otepa, Hao Island, French Polynesia | Mouse bioassay | [Chinain et al. 1999](#_bookmark33)  [12] |
|  | HO-91 | -  (fg CTX3C eq. cell^-1^) | NT | Otepa, Hao, French Polynesia | Receptor binding assay | Chinain et al 2010  [11] |
|  | MUR-4 | -  (fg CTX3C eq. cell^-1^) | NT | Moruroa, French Polynesia | Receptor binding assay | Chinain et al 2010  [11] |
|  |  |  |  |  |  |  |
| ***G. polynesiensis*** | CAWD212 | +  1,820  (fg CTX cell^-1^) | -  (fg MTX cell^-1^) | Rarotonga, Cook Islands, Pacific ocean | LC–MS/MS | Rhodes et al. 2014  [7] |
|  |  |  |  |  |  |  |
|  | MG-7 | +  17  (fg CTX3C eq. cell^-1^) | NT | Rikitea, Mangareva, French Polynesia | Receptor binding assay | Chinain et al 2010  [11] |
|  | RAI-1 | +  4,400  (fg CTX3C eq. cell^-1^) | NT | Motu Femme, Raivavae, French Polynesia | Receptor binding assay | Chinain et al 2010  [11] |
|  | RG-92 | +++  800  (×10^−4^ MU 1,000 cells−1) | +  0.06  (MU 1,000 cells^−1^) | Avatoru, Rangiroa Island, Tuamotu Archipelago, French Polynesia | Mouse bioassay | [Chinain et al. 1999](#_bookmark33)  [12] |
|  | RG-92 | +  2,800  (fg CTX3C eq. cell^-1^) | NT | Avatoru, Rangiroa Island, Tuamotu Archipelago, French Polynesia | Receptor binding assay | Chinain et al 2010  [11] |
|  | TB-92 | +++  1500  (×10^−4^ MU 1,000 cells^−1^) | +  0.1  (MU 1,000 cells^−1^) | Mataura, Tubuai Island, French Polynesia | Mouse bioassay | [Chinain et al. 1999](#_bookmark33)  [12] |
|  | TB-92 | +  4,300  (fg CTX3C eq. cell^-1^) | NT | Mataura, Tubuai Island, French Polynesia | Receptor binding assay | Chinain et al 2010  [11] |
|  |  |  |  |  |  |  |
| ***Gambierdiscus* ribotype 2** | CCMP 1655 (NCMA) | NT | +++ | Martinique, Caribbean, insular region of France | Hemolytic red blood cell assay | Holland et al. 2013  [1] |
|  | CCMP 1655 (NCMA) | +  1.3 to 8.7  (fg C-CTX1-eq. cell^-1^) | NT | Martinique, Caribbean, insular region of France | N2a cytotoxicity assay | Lartigue et al 2009  [16] |
|  | Mixed PR Gam 3 | +  1 | +  2 | Puerto Rico, USA | Fluorescent calcium flux assay | Lewis et al. 2016  [2] |
|  | Mixed PR Gam 3 | NT | +  p-MTX3 | Puerto Rico, USA | LC-LRMS/MS | Pisapia et al. 2017  [3] |
|  | Mixed PR Gam 4 | NT | +++ | Puerto Rico, USA | Hemolytic red blood cell assay | Holland et al. 2013  [1] |
|  | ST3 Gam F­2 | NT | +++ | St. Thomas, US Virgin Islands, USA | Hemolytic red blood cell assay | Holland et al. 2013  [1] |
|  | St. Maarten Gam 6 | +  2 | +  2 | St. Maarten, Caribbean, insular region of France | Fluorescent calcium flux assay | Lewis et al. 2016  [2] |
|  | St. Maarten Gam 10 | NT | +  p-MTX3 | St. Maarten, Collectivity of France, Caribbean | LC-LRMS/MS | Pisapia et al. 2017  [3] |
|  | SW Algae Gam1 | NT | +  p-MTX3 | South Water Cay, Belize | LC-LRMS/MS | Pisapia et al. 2017  [3] |
|  |  |  |  |  |  |  |
| ***G. scabrosus*** | KW070922_1 | 20  (×10^−4^ MU 1,000 cells^−1^) | 67  (×10^−4^ MU 1,000 cells^−1^) | Kashiwa-jima Island, Otsuki, Kochi, Japan | Mouse bioassay | Nishimura et al. 2013  [13] |
|  | KW070922_1 | ++  27.9 ± 3.8  (fg CTX3C eq. cell^-1^) | +  1.5 ± 0.2  (pg MTX eq. cell^−1^) | Kashiwa-jima Island, Otsuki, Kochi, Japan | N2a cytotoxicity assay | Pisapia et al. 2017  [10] |
|  | KW070922_1 | NT | - | Kashiwa-jima Island, Otsuki, Kochi, Japan | LC-LRMS/MS | Pisapia et al. 2017  [3] |
|  |  |  |  |  |  |  |
| ***G. silvae*** | UNR-30 | NT | +  p-MTX3 | Brazil | LC-LRMS/MS | Pisapia et al. 2017  [3] |
|  | VGO1167 | NT | +  p-MTX3 | Punta Hidalgo, Tenerife, Canary Islands | LC-LRMS/MS | Pisapia et al. 2017  [3] |
|  | VGO1167 | +  10.3 ± 2.7  (fg CTX3C eq. cell^-1^) | +  2.2 ± 0.3  (pg MTX eq. cell^−1^) | Punta Hidalgo, Tenerife, Canary Islands | N2a cytotoxicity assay | Pisapia et al. 2017  [10] |
|  | VGO1180 | NT | +  p-MTX3 | Punta Hidalgo, Tenerife, Canary Islands | LC-LRMS/MS | Pisapia et al. 2017  [3] |
|  | VGO1180 | +  12.4 ± 3.6  (fg CTX3C eq. cell^-1^) | +  3.3 ± 0.9  (pg MTX eq. cell^−1^) | Punta Hidalgo, Tenerife, Canary Islands | N2a cytotoxicity assay | Pisapia et al. 2017  [10] |
|  |  |  |  |  |  |  |
| ***Gambierdiscus* sp. (not all same species)** | RAI-2 | 102  (fg CTX3C eq. cell^-1^) | NT | Motu Femme, Raivavae, French Polynesia | Receptor binding assay | Chinain et al 2010  [11] |
|  | Viet Nam | + | NT | Cau Island, Binh Thuan, South China Sea, Viet Nam | LC-MS/MS | Roeder et al. 2010  [4] |
|  | Viet Nam | NT | +  p-MTX3 | Cau Island, Binh Thuan, South China Sea, Viet Nam | LC-LRMS/MS | Pisapia et al. 2017  [3] |
|  | Viet Nam | ++  40.8 ± 19.6  (fg CTX3C eq. cell^-1^) | +++  70.9 ± 44.8  (pg MTX eq. cell^−1^) | Cau Island, Binh Thuan, South China Sea, Viet Nam | N2a cytotoxicity assay | Pisapia et al. 2017  [10] |
|  |  |  |  |  |  |  |
| ***Gambierdiscus* sp. type 2** | M080828_2 | -  (×10^−4^ MU 1,000 cells^−1^) | -  (×10^−4^ MU 1,000 cells^−1^) | Muroto Promontory, Muroto City, Kochi, Japan | Mouse Bioassay | Nishimura et al. 2013  [13] |
|  | T070411_1 | -  (×10^−4^ MU 1,000 cells^−1^) | NT  (×10^−4^ MU 1,000 cells^−1^) | Tei Promontory, Konan City, Kochi, Japan | Mouse Bioassay | Nishimura et al. 2013  [13] |
|  |  |  |  |  |  |  |
| ***Gambierdiscus* sp. type 3** | WI9G | -  (×10^−4^ MU 1,000 cells^−1^) | -  (×10^−4^ MU 1,000 cells^−1^) | Itsumo, Kushimoto Town, Wakayama, Japan | Mouse Bioassay | Nishimura et al. 2013  [13] |
|  | WI11G | -  (×10^−4^ MU 1,000 cells^−1^) | ++  67  (×10^−4^ MU 1,000 cells^−1^) | Itsumo, Kushimoto Town, Wakayama, Japan | Mouse Bioassay | Nishimura et al. 2013  [13] |
|  |  |  |  |  |  |  |
| ***Gambierdiscus* sp. type 4** | IS00-01 | +  2.6  (fg CTX-1 eq. cell^-1^) | NT | Marakei, Republic of Kiribati, Pacific Ocean | CBA-N2a cytotoxicity assay | Xu et al. 2014  [21] |
|  |  |  |  |  |  |  |
|  | IS00-04 | +  4.4  (fg P-CTX-1 eq. cell^-1^) | NT | Marakei, Republic of Kiribati, Pacific Ocean | CBA-N2a cytotoxicity assay | Xu et al. 2014  [21] |
|  | ID0509-16 | +  4.1 (fg P-CTX-1 eq. cell^-1^) | NT | Marakei, Republic of Kiribati, Pacific Ocean | CBA-N2a cytotoxicity assay | Xu et al. 2014  [21] |
|  | ID0510-22 | +  6.0  (fg P-CTX-1 eq. cell^-1^) | NT | Marakei, Republic of Kiribati, Pacific Ocean | CBA-N2a cytotoxicity assay | Xu et al. 2014  [21] |
|  |  |  |  |  |  |  |
| ***Gambierdiscus* sp. type 5** | DS0511-03 | +  0.010  (fg P-CTX-1 eq. cell^-1^) | NT | Marakei, Republic of Kiribati, Pacific Ocean | CBA-N2a cytotoxicity assay | Xu et al. 2014  [21] |
|  | M4SWC11 | -  (fg P-CTX-1 eq cell^-1^) | NT | Marakei Island, Kiribati | N2a cytotoxicity assay | Dai et al. 2017  [14] |
|  |  |  |  |  |  |  |
| ***G. toxicus*** | GTT-91 | -  (×10^−4^ MU 1,000 cells^−1^) | 0.7  (MU 1,000 cells−1) | Teahupoo, Tahiti, French Polynesia | Mouse bioassay | [Chinain et al., 1999](#_bookmark33)  [12] |
|  | GTT-91 | -  (fg CTX3C eq. cell^-1^) | NT | Teahupoo, Tahiti, French Polynesia | Receptor binding assay | Chinain et al 2010  [11] |
|  | GTT-91 | NT | +  p-MTX3 | Teahupoo, Tahiti, French Polynesia | LC-LRMS/MS | Pisapia et al. 2017  [3] |
|  | HIT-0 | NT | +  p-MTX3 | Hitiaa, Tahiti, French Polynesia | LC-LRMS/MS | Pisapia et al. 2017  [3] |
|  | HIT-0 | -  (pg CTX3C eq. cell^-1^) | NT | Hitiaa, Tahiti, French Polynesia | Receptor binding assay | Chinain et al 2010  [11] |
|  | HIT-25 | +  22  (fg CTX3C eq. cell^-1^) | NT | Hitiaa, Tahiti, French Polynesia | Receptor binding assay | Chinain et al 2010  [11] |
|  | HIT-25 | NT | +  p-MTX3 | Hitiaa, Tahiti, French Polynesia | LC-LRMS/MS | Pisapia et al. 2017  [3] |
|  | PAP-1 | +  28  (fg CTX3C eq. cell^-1^) | NT | Papara, Tahiti, French Polynesia | Receptor binding assay | Chinain et al 2010  [11] |
|  | REN-1 | -  (×10^−4^ MU 1,000 cells^−1^) | 1.7  (MU 1,000 cells^−1^) | Saint-Leudville fringing reef, La Réunion Island, Indian Ocean | Mouse bioassay | [Chinain et al., 1999](#_bookmark33)  [12] |
|  | REN-1 | -  (fg CTX3C eq. cell^-1^) | NT | Saint-Leudville fringing reef, La Réunion Island, Indian Ocean | Receptor binding assay | Chinain et al 2010  [11] |
|  | TUR | -  (×10^−4^ MU 1,000 cells^−1^) | 0.6  (MU 1,000 cells^−1^) |  | Mouse bioassay | [Chinain et al., 1999](#_bookmark33)  [12] |
|  |  |  |  |  |  |  |
| **Unidentified *Gambierdiscus* isolates*** | 135 | ? | ++ | Bermuda, Caribbean | Mouse bioassay | [Bomber et al. 1989](#_bookmark28) [23] |
|  | 157 | ? | + | Drift algae, Caribbean | Mouse bioassay | [Bomber et al. 1989](#_bookmark28)  [23] |
|  | 158 | ? | ++ | Drift algae, Caribbean | Mouse bioassay | [Bomber et al. 1989](#_bookmark28)  [23] |
|  | 162 | ? | ++ | Gingerbread  Grounds, Bahamas, Atlantic Ocean | Mouse bioassay | [Bomber et al. 1989](#_bookmark28)  [23, 24] |
|  | 163 | ? | +++ | Gingerbread  Grounds, Bahamas, Atlantic Ocean | Mouse bioassay | [Bomber et al. 1989](#_bookmark28)  [23, 24] |
|  | 165 | ? | +++ | Drift algae, Caribbean | Mouse bioassay | [Bomber et al. 1989](#_bookmark28)  [23] |
|  | 169 | ? | ++ | Drift algae, Caribbean | Mouse bioassay | [Bomber et al. 1989](#_bookmark28)  [23] |
|  | 170 | ? | +++ | Gingerbread  Grounds, Bahamas, Atlantic Ocean | Mouse bioassay | [Bomber et al. 1989](#_bookmark28)  [23, 24] |
|  | 171 | ? | ++ | Gingerbread  Grounds, Bahamas, Atlantic Ocean | Mouse bioassay | [Bomber et al., 1989](#_bookmark28)  [23, 24] |
|  | 172 | ? | ++ | Gingerbread  Grounds, Bahamas, Atlantic Ocean | Mouse bioassay | [Bomber et al., 1989](#_bookmark28)  [23, 24] |
|  | 175 | ? | +++ | Martinique, Caribbean | Mouse bioassay | [Bomber et al., 1989](#_bookmark28)  [23] |
|  | 177 | ? | +++ | Hawaii, USA | Mouse bioassay | [Bomber et al., 1989](#_bookmark28)  [23] |
|  | 196 | ? | ++ | Florida Keys, USA | Mouse bioassay | [Bomber et al., 1989](#_bookmark28)  [23] |
|  | 199 | ? | +++ | Florida Keys, USA | Mouse bioassay | [Bomber et al., 1989](#_bookmark28)  [23] |
|  | 200 | ? | ++ | Florida Keys, USA | Mouse bioassay | [Bomber et al., 1989](#_bookmark28)  [23] |
|  | 300 | ? | +++ | Florida Keys, USA | Mouse bioassay | [Bomber et al., 1989](#_bookmark28)  [23] |
|  | 350 | ? | +++ | Atlantic, Caribbean | Mouse bioassay | [Bomber et al., 1989](#_bookmark28)  [23] |
|  | 94-13 | - | - | Southwest Puerto Rico, USA | Mouse bioassay | [Tosteson et al. 1989](#_bookmark81)  [23] |
|  | 101-16 | - | - | Southwest Puerto Rico, USA | Mouse bioassay | [Tosteson et al. 1989](#_bookmark81)  [23] |
|  | 105-13 | - | + | Southwest Puerto Rico, USA | Mouse bioassay | [Tosteson et al. 1989](#_bookmark81)  [23] |
|  | 106-13 | - | - | Southwest Puerto Rico, USA | Mouse bioassay | [Tosteson et al. 1989](#_bookmark81)  [23] |
|  | 107-24 | - | - | Southwest Puerto Rico, USA | Mouse bioassay | [Tosteson et al. 1989](#_bookmark81)  [23] |
|  | B6 | ? | + | Gingerbread  Grounds, Bahamas, Atlantic Ocean | Mouse bioassay | [Bomber et al., 1989](#_bookmark26)  [23, 24] |
|  | B30 | ? | + | Gingerbread  Grounds, Bahamas, Atlantic Ocean | Mouse bioassay | [Bomber et al. 1989](#_bookmark26)  [23, 24] |
|  | Cd4 | ? | +++ | Knight Key, Florida, USA | Mouse bioassay | Babinchak et al. 1986  [25] |
|  | Cd8 | ? | ++ | Knight Key, Florida, USA | Mouse bioassay | Babinchak et al. 1986  [23] |
|  | Cd9 | ? | + | Knight Key, Florida, USA | Mouse bioassay | Babinchak et al. 1986  [23] |
|  | Cd10 | ? | ++ | Knight Key, Florida, USA | Mouse bioassay | Babinchak et al. 1986  [23] |
|  | Cd13 | ? | + | Knight Key, Florida, USA | Mouse bioassay | Babinchak et al. 1986  [23] |
|  | Cd20 | ? | +++ | Knight Key, Florida, USA | Mouse bioassay | Babinchak et al. 1986  [23] |
|  | CGT-a | + | + | Gambier Island, French Polynesia | Mouse bioassay | [Bagnis et al. 1980](#_bookmark19)  [23] |
|  | CGT-b | ++ | + | Gambier Island, French Polynesia | Mouse bioassay | [Bagnis et al. 1980](#_bookmark19) [77] |
|  | Clone 1 | +++ | + | Gambier Island, French Polynesia | Mouse bioassay | [Durand-Clément 1987](#_bookmark40)  [23] |
|  | Clone 2 | ++ | + | Gambier Island, French Polynesia | Mouse bioassay | [Durand-Clément 1987](#_bookmark40)  [26] |
|  | Clone 3 | ++ | + | Gambier Island, French Polynesia | Mouse bioassay | [Durand-Clément, 1987](#_bookmark40)  [26] |
|  | Clone 4 | ++ | + | Gambier Island, French Polynesia | Mouse bioassay | [Durand-Clément 1987](#_bookmark40)  [26] |
|  | Clone 5 | ++ | + | Gambier Island, French Polynesia | Mouse bioassay | [Durand-Clément 1987](#_bookmark40)  [26] |
|  | Culture 1 | + | + | Gambier Island, French Polynesia | Mouse bioassay | [Yasumoto et al. 1979](#_bookmark90)  [27] |
|  | FP | - | NT | Gambier Island, French Polynesia | Mouse bioassay | [Holmes et al. 1991](#_bookmark50)  [28] |
|  | GT178 | - | NT | Kahala Bay, Hawaii, USA | Mouse bioassay | [Holmes et al. 1991](#_bookmark50)  [28] |
|  | GT18/1 | - | NT | Kahala Bay, Hawaii, USA | Mouse bioassay | [Holmes et al. 1991](#_bookmark50)  [28] |
|  | GT360 | - | NT | South Sound, US Virgin Islands, USA | Mouse bioassay | [Holmes et al. 1991](#_bookmark50)  [28] |
|  | HIMB | + | ++ | Northwest Hawaiian Islands, USA | Mouse bioassay | [Withers 1984](#_bookmark84)  [29] |
|  | HR1 | - | NT | Hoffman Rocks, Queensland, Australia | Mouse bioassay | [Holmes et al. 1991](#_bookmark50)  [28] |
|  | HR7 | - | NT | Hoffman Rocks, Queensland, Australia | Mouse bioassay | [Holmes et al. 1991](#_bookmark50)  [28] |
|  | MQ-1 | ? | + | Florida Keys, USA | Mouse bioassay | [Bomber et al. 1988b](#_bookmark26)  [30] |
|  | NQ1 | - | NT | Hastings Reef, Queensland, Australia | Mouse bioassay | [Holmes et al. 1991](#_bookmark50)  [28] |
|  | NQ1 | - | NT | Hastings Reef, , Queensland, Australia | Mouse bioassay | [Holmes et al. 1991](#_bookmark50)  [28] |
|  | NQ1 | - | NT | Hastings Reef, Queensland, Australia | Mouse bioassay | [Holmes et al. 1991](#_bookmark50)  [28] |
|  | NQ2/7 | + | NT | Arlington reef, Queensland, Australia | Mouse bioassay | [Holmes et al. 1991](#_bookmark50)  [28] |
|  | SQ3/3 | - | NT | Flinders Reef, Queensland, Australia | Mouse bioassay | [Holmes et al. 1991](#_bookmark50)  [28] |
|  | Strain A | ++ | + | Gambier Island, French Polynesia | Mouse bioassay | [Durand-Clément 1987](#_bookmark40)  [26] |
|  | Strain B | +++ | + | Gambier Island, French Polynesia | Mouse bioassay | [Durand-Clément 1987](#_bookmark40)  [26] |
|  | T39 =  CCMP 1553 | ? | +++ | Tern Island, Hawaii, USA | Mouse bioassay | [Babinchak et al. 1986](#_bookmark15)  [25] |
|  | WC1/1 | + | NT | Platypus Bay, Station 1, Queensland, Australia | Mouse bioassay | [Holmes et al. 1991](#_bookmark50)  [28] |
|  | WC1/1 | + | NT | Platypus Bay, Station 1, Queensland, Australia | Mouse bioassay | [Holmes et al. 1991](#_bookmark50)  [28] |
|  | WC1/18 | - | NT | Platypus Bay, Station 1, Queensland, Australia | Mouse bioassay | [Holmes et al. 1991](#_bookmark50)  [28] |
|  | WC2/1 | - | NT | Platypus Bay, Station 2, Queensland, Australia | Mouse bioassay | [Holmes et al. 1991](#_bookmark50)  [28] |
|  | WC3/1 | - | NT | Platypus Bay, Station 1, Queensland, Australia | Mouse bioassay | [Holmes et al. 1991](#_bookmark50)  [28] |
|  | T39 | ? | +++ | Tern Island, Hawaii, USA | Mouse bioassay | [Babinchak et al. 1986](#_bookmark15)  [25] |

********Species identity unknown because toxicity testing occurred prior to development of accurate molecular identification methods.*

References

1. Holland WC, Litaker RW, Tomas CR, Kibler SR, Place AR, Davenport ED, et al. Differences in the toxicity of six *Gambierdiscus* (Dinophyceae) species measured using an in vitro human erythrocyte lysis assay. Toxicon. 2013;65: 15-33. doi: 10.1016/j.toxicon.2012.12.016. PubMed PMID: WOS:000317172200004.

2. Lewis RJ, Inserra M, Vetter I, Holland WC, Hardison DR, Tester PA, et al. Rapid extraction and identification of maitotoxin and ciguatoxin-like toxins from Caribbean and Pacific *Gambierdiscus* using a new functional bioassay. PLoS One. 2016;Jul 28;11(7):e0160006. doi: 10.1371/journal.pone.0160006. PubMed Central PMCID: PMC4965106

3. Pisapia F, Sibat M, Herrenknecht C, Lhaute K, Gaiani G, Ferron P-J, et al. Maitotoxin-4, a Novel MTX Analog Produced by *Gambierdiscus excentricus*. Mar Drugs. 2017;Jul 11;15(7). pii: E220. doi: 10.3390/md15070220. PubMed Central PMCID: PMC PMC5532662.

4. Roeder K, Erler K, Kibler S, Tester P, Ho VT, Lam NN, et al. Characteristic profiles of ciguatera toxins in different strains of *Gambierdiscus* spp. Toxicon. 2010;56(5): 731-8. doi: 10.1016/j.toxicon.2009.07.039. PubMed PMID: WOS:000281499600009.

5. Laza-Martinez A, David H, Riobo P, Miguel I, Orive E. Characterization of a strain of *Fukuyoa paulensis* (Dinophyceae) from the Western Mediterranean Sea. J Eukaryot Microbiol. 2016;63(4): 481-97. doi: 10.1111/jeu.12292. PubMed PMID: WOS:000379616400007.

6. Holmes MJ. *Gambierdiscus yasumotoi* sp. nov. (Dinophyceae), a toxic benthic dinoflagellate from southeastern Asia. J Phycol. 1998;34(4): 661-8. doi: 10.1046/j.1529-8817.1998.340661.x. PubMed PMID: WOS:000075712400014.

7. Rhodes L, Harwood T, Smith K, Argyle P, Munday R. Production of ciguatoxin and maitotoxin by strains of *Gambierdiscus australes*, *G. pacificus* and *G. polynesiensis* (Dinophyceae) isolated from Rarotonga, Cook Islands. Harmful Algae. 2014;39: 185-90. doi: 10.1016/j.hal.2014.07.018. PubMed PMID: WOS:000345469100021.

8. Rhodes LL, Smith KF, Verma A, Murray S, Harwood DT, Trnski T. The dinoflagellate genera *Gambierdiscus* and *Ostreopsis* from subtropical Raoul Island and North Meyer Island, Kermadec Islands. New Zealand Journal of Marine and Freshwater Research. 2016. doi: 10.1080/00288330.2016.1270337

9. Rhodes LL, Smith KF, Murray S, Harwood DT, Trnski T, Munday R. The epiphytic genus *Gambierdiscus* (Dinophyceae) in the Kermadec Islands and Zealandia Regions of the southwestern Pacific and the associated risk of cguatera fsh poisoning. Mar Drugs. 2017;15: 219. doi: 10.3390/md15070219.

10. Pisapia F, Holland WC, Hardison DR, Litaker RW, Fraga S, Nishimura T, et al. Toxicity screening of 13 *Gambierdiscus* strains using neuro-2a and erythrocyte lysis bioassays. Harmful Algae. 2017;63: 173-83. doi: 10.1016/j.hal.2017.02.005. PubMed PMID: WOS:000399849100018.

11. Chinain M, Darius HT, Ung A, Cruchet P, Wang ZH, Ponton D, et al. Growth and toxin production in the ciguatera-causing dinoflagellate *Gambierdiscus polynesiensis* (Dinophyceae) in culture. Toxicon. 2010;56(5): 739-50. doi: 10.1016/j.toxicon.2009.06.013. PubMed PMID: WOS:000281499600010.

12. Chinain M, Faust MA, Pauillac S. Morphology and molecular analyses of three species of *Gambierdiscus* (Dinophceae): *G. pacificus*, sp. nov., *G. australes*, sp. nov., and *G. polynesiensis*, sp. nov. J Phycol. 1999;35: 1282-96.

13. Nishimura T, Sato S, Tawong W, Sakanari H, Uehara K, Shah MMR, et al. Genetic diversity and distribution of the ciguatera-causing dinoflagellate *Gambierdiscus* spp. (Dinophyceae) in coastal areas of Japan. PLoS ONE. 2013;8(4): 14. doi: 10.1371/journal.pone.0060882. PubMed PMID: WOS:000317383200024.

14. Dai X, Mak YL, Lu CK, Mei HH, Wu JJ, Lee WH, et al. Taxonomic assignment of the benthic toxigenic dinoflagellate *Gambierdiscus* sp. type 6 as *Gambierdiscus balechii* (Dinophyceae), including its distribution and ciguatoxicity. Harmful Algae. 2017;67: 107-18. Epub 2017/08/02. doi: 10.1016/j.hal.2017.07.002. PubMed PMID: 28755713.

15. Fraga S, Rodríguez F, Riobó P, Bravo I. *Gambierdiscus balechii* sp. nov (Dinophyceae), a new benthic toxic dinoflagellate from the Celebes Sea (SW Pacific Ocean). Harmful Algae. 2016;58: 93–105.

16. Lartigue J, Jester ELE, Dickey RW, Villareal TA. Nitrogen source effects on the growth and toxicity of two strains of the ciguatera-causing dinoflagellate *Gambierdiscus toxicus*. Harmful Algae. 2009;8(5): 781-91. doi: 10.1016/j.hal.2008.05.006. PubMed PMID: WOS:000267153900020.

17. Smith KF, Rhodes L, Verma A, Curley BG, Harwood DT, Kohli GS, et al. A new *Gambierdiscus* species (Dinophyceae) from Rarotonga, Cook Islands: *Gambierdiscus cheloniae* sp. nov. Harmful Algae. 2016;60: 45-56. doi: http://dx.doi.org/10.1016/j.hal.2016.10.006.

18. Fraga S, Rodriguez F, Caillaud A, Diogene J, Raho N, Zapata M. *Gambierdiscus excentricus* sp. nov. (Dinophyceae), a benthic toxic dinoflagellate from the Canary Islands (NE Atlantic Ocean). Harmful Algae. 2011;11: 10-22. doi: 10.1016/j.hal.2011.06.013. PubMed PMID: WOS:000297872600002.

19. Rhodes L, Smith KF, Verma A, Curley BG, Harwood DT, Murray S, et al. A new species of Gambierdiscus (Dinophyceae) from the south-west Pacific: *Gambierdiscus honu* sp. nov. Harmful Algae. 2017;65: 61-70. doi: 10.1016/j.hal.2017.04.010. PubMed PMID: WOS:000402445000007.

20. Kretzschmar AL, Verma A, Harwood T, Hoppenrath M, Murray S. Characterization of *Gambierdiscus lapillus* sp. nov. (Gonyaulacales, Dinophyceae): a new toxic dinoflagellate from the Great Barrier Reef (Australia). J Phycol. 2017;53(2): 283-97. doi: 10.1111/jpy.12496.

21. Xu YX, Richlen ML, Morton SL, Mak YL, Chan LL, Tekiau A, et al. Distribution, abundance and diversity of *Gambierdiscus* spp. from a ciguatera-endemic area in Marakei, Republic of Kiribati. Harmful Algae. 2014;34: 56-68. doi: 10.1016/j.hal.2014.02.007. PubMed PMID: WOS:000335276300007.

22. Caillaud A, de la Iglesia P, Barber E, Eixarch H, Mohammad-Noor N, Yasumoto T, et al. Monitoring of dissolved ciguatoxin and maitotoxin using solid-phase adsorption toxin tracking devices: Application to *Gambierdiscus pacificus* in culture. Harmful Algae. 2011;10(5): 433-46. doi: 10.1016/j.hal.2011.02.004. PubMed PMID: WOS:000292233300003.

23. Bomber JW, Tindall DR, Miller DM. Genetic variability in toxin potencies among seventeen clones of *Gambierdiscus toxicus* (Dinophyceae). J Phycol. 1989;25(4): 617-25. PubMed PMID: WOS:A1989CG87500001.

24. Parsons ML, Richlen ML, editors. An overview of ciguatera fish poisoning in the Bahamas. The 15th Symposium on the Natural History of the Bahamas; 2016; San Salvador Bahamas: Gerace Research Centre.

25. Babinchak JA, Jollow DJ, Voegtline MS, Higerd TB. Toxin production by *Gambierdiscus toxicus* isolated from the Florida Keys. Mar Fish Rev. 1986;48(4): 53-6. PubMed PMID: ISI:A1986J809900012.

26. Durand-Clément M. Study of production and toxicity of cultured *Gambierdiscus toxicus*. Biological Bulletin. 1987;172(1): 108-21. doi: 10.2307/1541610. PubMed PMID: WOS:A1987G373600009.

27. Yasumoto T, Nakajima I, Oshima Y, Bagnis R. A new toxic dinoflagellate found in association with ciguatera. In: Taylor DL, Seliger HH, editors. Toxic Dinoflagellate Blooms. New York: Elsevier North Holland, Inc.; 1979. p. 65-70.

28. Holmes MJ, Lewis RJ, Poli MA, Gillespie NC. Strain dependent production of ciguatoxin precursors (Gambiertoxins) by *Gambierdiscus toxicus* (Dinophyceae) in culture. Toxicon. 1991;29(6): 761-75.

29. Withers NW. Ciguatera research in the northwestern Hawaiian Islands: Laboratory and field studies on ciguatoxigenic dinoflagellates in the Hawaiian Archipelago. In: Grigg RW, Pfund RT, editors. Proceedings of the Second Symposium on Resource Investigations in the Northwestern Hawaiian Islands. 1: University of Hawaii Sea Grant; 1984. p. 144-56.

30. Bomber JW, Tindall DR. Genetic variability in acclimated cell toxicities among Atlantic and Pacific clones of the ciguatera causing dinoflagellate *Gambierdiscus toxicus*. FASEB J. 1988;2(4): A375-A. PubMed PMID: WOS:A1988M612100461.
