# Supplementary material for: Ciguatoxicity of Gambierdiscus and Fukuyoa species from the Caribbean and Gulf of Mexico
Source: PLoS One. 2017 Oct 18;12(10):e0185776. doi: 10.1371/journal.pone.0185776 (PMC5646788; doi:10.1371/journal.pone.0185776)
Supplement: S2 Table — (DOCX) [file pone.0185776.s002.docx]

**S2 Table. Comparison of the *Gambierdiscus* and *Fukuyoa*** **growth rate estimates determined in this study versus rates published in other studies**. The table is divided into upper section where growth rates were estimated using direct cell counts and those with used relative changes in chlorophyll *a* fluorescence to estimate growth.

|  | **Growth Rates (d^-1^) ± standard deviation** | | | | | | | |
| --- | --- | --- | --- | --- | --- | --- | --- | --- |
| **Study** | ***G. belizeanus*** | ***G. caribaeus*** | ***G. carolinianus*** | ***G. carpenteri*** | ***Gambierdiscus***  **ribotype II** | ***G. silvae*** | ***G. excentricus*** | ***F. ruetzleri*** |
| **Growth estimated based on direct cell counts** |  |  |  |  |  |  |  |  |
| This study | 0.167 ± 0.026 | 0.172 ± 0.024 | 0.174 ± 0.017 | 0.163 ± 0.026 | 0.128 ± 0.010 | 0.09 | 0.05 | 0.176 ± 0.003 |
| Lartigue et al. 2009 [1] |  | 0.14 |  |  | 0.11 |  |  |  |
| Parsons et al. 2010 [2] |  | 0.110-0.156 |  |  |  |  |  |  |
| Chinain et al. 2010 [3] | 0.140 |  |  |  |  |  |  |  |
|  |  |  |  |  |  |  |  |  |
| **Growth estimated by changes in Chl *a* fluorescence** |  |  |  |  |  |  |  |  |
| Kibler et al. 2015 [4] | 0.146 | 0.194 | 0.159 |  | 0.104 |  |  | 0.208 |
| Xu et al. 2016 [5] | 0.191-0.235 | 0.204-0.229 | 0.152 – 0.191 | 0.132-0.201 |  | ~0.124 |  |  |

References

1. Lartigue J, Jester ELE, Dickey RW, Villareal TA. Nitrogen source effects on the growth and toxicity of two strains of the ciguatera-causing dinoflagellate *Gambierdiscus toxicus*. Harmful Algae. 2009;8(5): 781-91. doi: 10.1016/j.hal.2008.05.006. PubMed PMID: WOS:000267153900020.

2. Parsons ML, Settlemier CJ, Bienfang PK. A simple model capable of simulating the population dynamics of *Gambierdiscus*, the benthic dinoflagellate responsible for ciguatera fish poisoning. Harmful Algae. 2010;10(1): 71-80. doi: 10.1016/j.hal.2010.07.002. PubMed PMID: WOS:000283965700009.

3. Chinain M, Darius HT, Ung A, Cruchet P, Wang ZH, Ponton D, et al. Growth and toxin production in the ciguatera-causing dinoflagellate *Gambierdiscus polynesiensis* (Dinophyceae) in culture. Toxicon. 2010;56(5): 739-50. doi: 10.1016/j.toxicon.2009.06.013. PubMed PMID: WOS:000281499600010.

4. Kibler SR, Tester PA, Kunkel KE, Moore SK, Litaker RW. Effects of ocean warming on growth and distribution of dinoflagellates associated with ciguatera fish poisoning in the Caribbean. Ecol Model. 2015;316: 194-210. doi: 10.1016/j.ecolmodel.2015.08.020. PubMed PMID: WOS:000364248600019.

5. Xu YX, Richlen ML, Liefer JD, Robertson A, Kulis D, Smith TB, et al. Influence of environmental variables on *Gambierdiscus* spp. (Dinophyceae) growth and distribution. PLOS ONE. 2016;11(4): 30. doi: 10.1371/journal.pone.0153197. PubMed PMID: WOS:000374131200059.
