# Supplementary material for: Ciguatoxicity of Gambierdiscus and Fukuyoa species from the Caribbean and Gulf of Mexico
Source: PLoS One. 2017 Oct 18;12(10):e0185776. doi: 10.1371/journal.pone.0185776 (PMC5646788; doi:10.1371/journal.pone.0185776)
Supplement: S3 Table — (DOCX) [file pone.0185776.s003.docx]

Table S3. A. Ratio of the highest toxicity isolate divided by the lowest isolate in fg CTX3C eq. cell^-1^. *One of the *G. carolinianus* isolates did not have detectible toxin so value of 0.05 fg CTX3C eq. cell^-1^, which is below the limit of detection was used to estimate the ratio.

|  | *min* | *Max* | *Ratio Max/ Min* |
| --- | --- | --- | --- |
| *F. ruetzleri* | 0.9 | 24.5 | 27.2 |
| *G. belizeanus* | 0.4 | 2.5 | 6.3 |
| *G. caribaeus* | 0.2 | 1.3 | 6.5 |
| *G. carolinianus** | 0.05 | 1 | 20.0 |
| *G. carpenteri* | 0.3 | 1.4 | 4.7 |
| *Gambierdiscus ribotype 2* | 4.7 | 10.9 | 2.3 |

B. Ratio of mean among species toxicity (fg CTX3C eq. cell^-1^). In case of *G. excentricus* and *G. silvae* the estimate for the single clone measured was used to represent the mean value.

|  | A | *F. ruetzleri* | *G. belizeanus* | *G. caribaeus* | *G. carolinianus* | *G. carpenteri* | *G. excentricus* | *Gambierdiscus* ribotype 2 | *G. silvae* |
| --- | --- | --- | --- | --- | --- | --- | --- | --- | --- |
|  |  | Ratio A/B | Ratio A/B | Ratio A/B | Ratio A/B | Ratio A/B | Ratio A/B | Ratio A/B | Ratio A/B |
| B | Mean | 10.6 | 0.9 | 0.7 | 0.3 | 0.89 | 469.0 | 6.62 | 19.6 |
| *F. ruetzleri* | 10.6 | 1.0 | 0.08 | 0.06 | 0.03 | 0.08 | 44.3 | 0.62 | 1.9 |
| *G. belizeanus* | 0.85 |  | 1.0 | 0.8 | 0.3 | 1.05 | 551.8 | 7.79 | 23.0 |
| *G. caribaeus* | 0.66 |  |  | 1.00 | 0.4 | 1.35 | 710.6 | 10.03 | 29.7 |
| *G. carolinianus* | 0.27 |  |  |  | 1.0 | 3.30 | 1737.0 | 24.52 | 72.6 |
| *G. carpenteri* | 0.89 |  |  |  |  | 1.00 | 527.0 | 7.44 | 22.0 |
| *G. excentricus* | 469 |  |  |  |  |  | 1.00 | 0.01 | 0.04 |
| *Gambierdiscus* ribotype 2 | 6.62 |  |  |  |  |  |  | 1.00 | 3.0 |
| *G. silvae* | 19.6 |  |  |  |  |  |  |  | 1.00 |
