# Supplementary figures and images for: Ciguatoxicity of Gambierdiscus and Fukuyoa species from the Caribbean and Gulf of Mexico
Source: PLoS One. 2017 Oct 18;12(10):e0185776. doi: 10.1371/journal.pone.0185776 (PMC5646788; doi:10.1371/journal.pone.0185776)

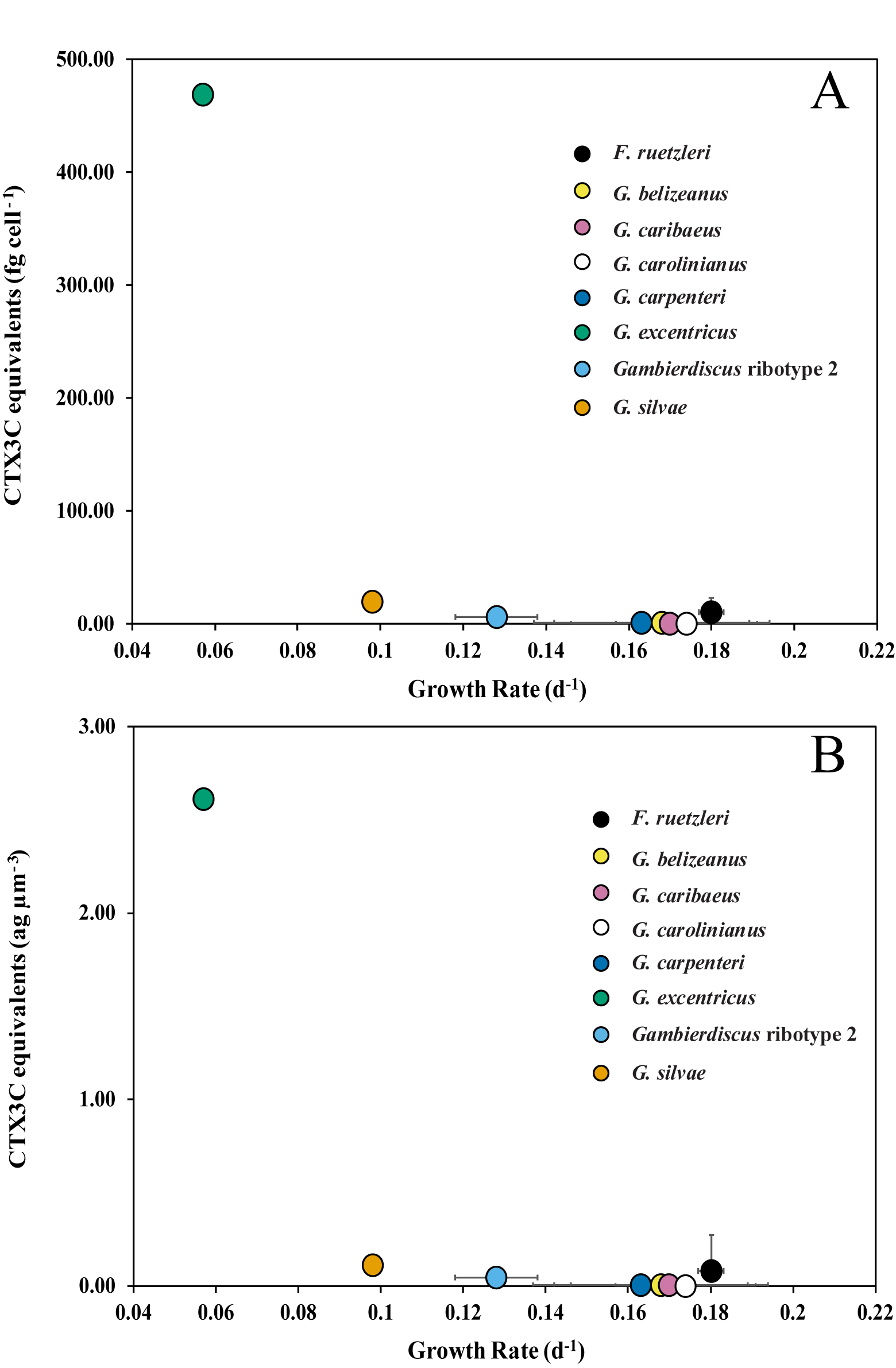

Supplement: S1 Fig — Cellular toxicity versus growth rate for each of the Gambierdiscus and Fukuyoa species normalized (A) to femtograms (fg) CTX3C eq. cell-1 and (B) attograms (ag) CTX3C eq. per μm-3 biovolume. Error bars = ± 1 standard deviation. This graph visually demonstrates the large difference in variation in toxicity of G. excentricus relative to the other species. (TIF) [file pone.0185776.s004.tif]
